# Supplementary material for: Divergence of trafficking and polarization mechanisms for PIN auxin transporters during land plant evolution
Source: Plant Commun. 2023 Jul 31;5(1):100669. doi: 10.1016/j.xplc.2023.100669 (PMC10811345; doi:10.1016/j.xplc.2023.100669)
Supplement: Document S1. Supplemental Figures 1–9 and Supplemental Tables 1 and 2 [file mmc1.pdf]

Plant Communications, Volume *5*

Supplemental information

Divergence of trafficking and polarization mechanisms for PIN auxin transporters during land plant evolution

Han Tang, Kuan-Ju Lu, YuZhou Zhang, You-Liang Cheng, Shih-Long Tu, and Jiří Friml

Supplemental Information

Divergence of trafficking and polarization mechanisms for PIN auxin transporters during land plant evolution

Han Tang[1,5,6], Kuan-Ju Lu[2,6], YuZhou Zhang[3], You-Liang Cheng[4], Shih-Long Tu[4], and Jiří Friml[1,*]

1. Institute of Science and Technology Austria (ISTA), Am Campus 1, 3400 Klosterneuburg, Austria
2. Graduate Institute of Biotechnology, National Chung Hsing University, No. 145, Xingda Rd., South Dist., Taichung 40227, Taiwan, R.O.C.
3. College of Life Sciences, Northwest A&F University, Shaanxi, Yangling, China.
4. Institute of Plant and Microbial Biology, Academia Sinica, 128 Sec. 2, Academia Rd, Nankang, Taipei 11529, Taiwan, R.O.C.
5. Current address: Graduate Institute of Biochemistry, National Chung Hsing University, No. 145, Xingda Rd., South Dist., Taichung 40227, Taiwan, R.O.C.

6. These authors contribute equally to this work.

* Correspondence: jiri.friml@ist.ac.at, +43 2243 9000 5401

The supplemental file includes 9 supplemental figures and 2 supplemental tables

Supplemental figure 1. Identity index of all selected PINs compared to AtPIN1.

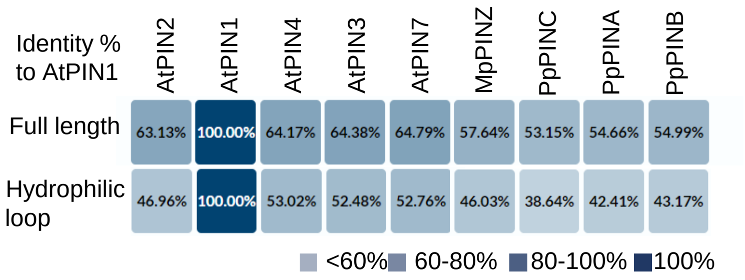

Supplemental figure 1. Identity index of all selected PINs compared to AtPIN1.

Alignment of full length sequence and hydrophilic loop region for canonical PINs from *Physcomitrium patens* (Pp), *Marchantia polymorpha* (Mp), and *Arabidopsis thaliana* (At).

Supplemental figure 2. PIN structures predicted by Alphafold2 algorism.

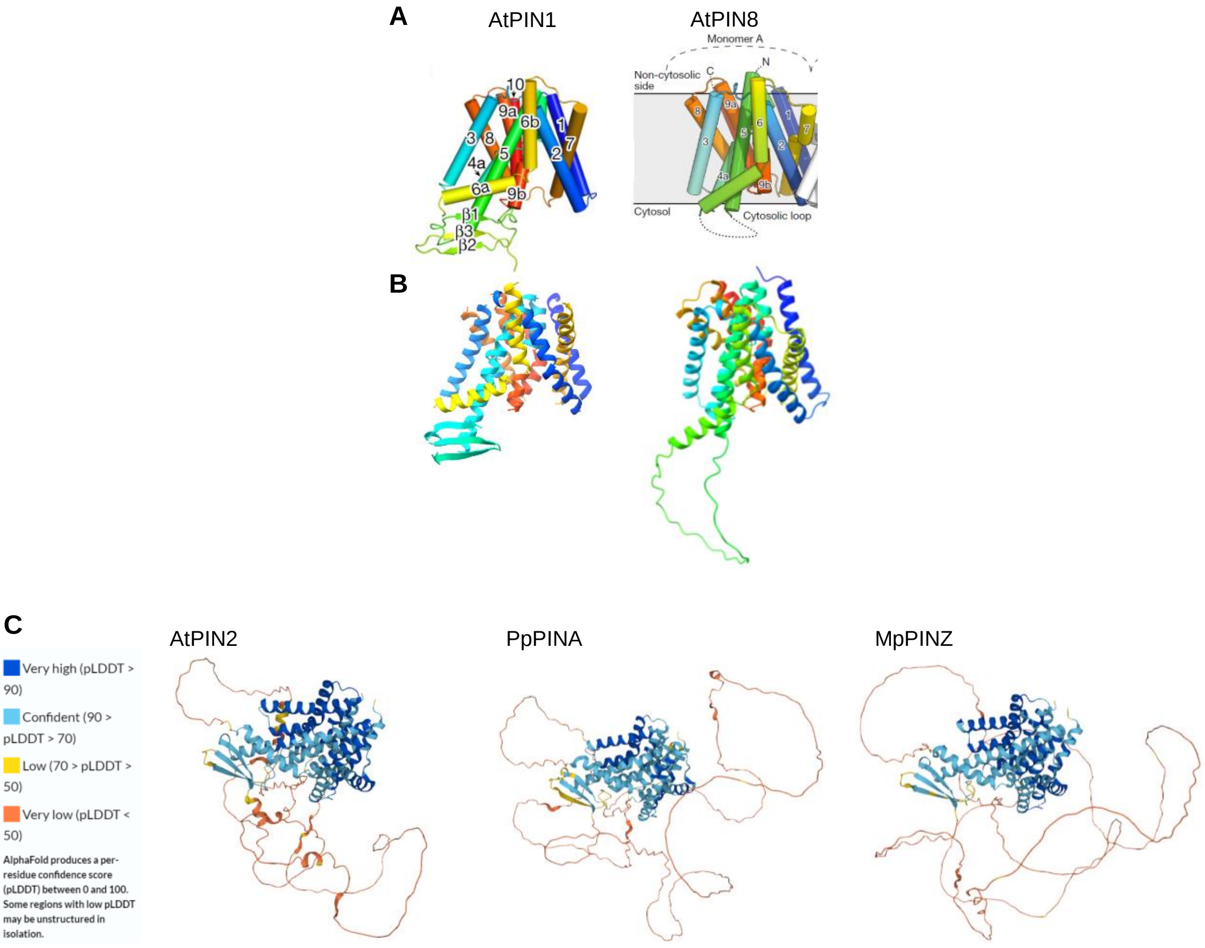

Supplemental figure 2. PIN structures predicted by Alphafold2 algorism.

(A) Overall structures of AtPIN1 and AtPIN8 monomer modified from Ung *et al*. and Yang *et al*. (Ung *et al.,* 2022; Yang *et al.,* 2022)
(B) Predicted structures of AtPIN1 and AtPIN8 by AlphaFold2.
(C) Predicted structures of AtPIN2, PpPINA, and MpPINZ with the confidence score provided by AlphaFold2.

Supplemental figure 3. Genotype confirmation of transgenic lines.

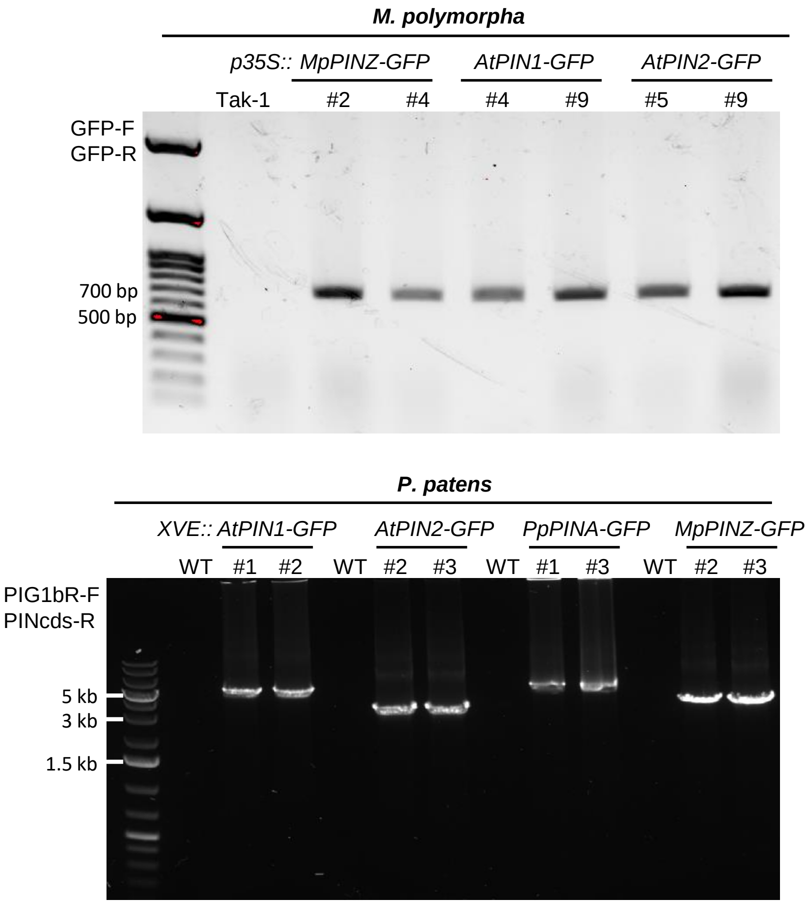

Supplemental figure 3. Confirmation of transgenic lines.

Genomic DNAs extracted from indicated transgenic lines were used as templates for PCR reactions. Primer sets used in the PCR were indicated in the left, and the primer sequences were shown in supplemental table. Two independent lines for each construct were confirmed.

Supplemental figure 4. PpPINA is evenly distributed on the plasma membrane in moss leaves near the base.

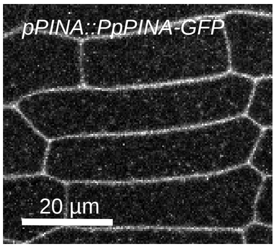

Supplemental figure 4. PpPINA is evenly distributed on the plasma membrane in moss leaves near the base.

As previously reported, PpPINA-GFP showed symmetric distribution in gametophytic leaves. (Viaene *et al.,* 2014).

Supplemental figure 5. PpPINB is evenly distributed on the plasma membrane with a high cytosolic signal in moss protonema cells.

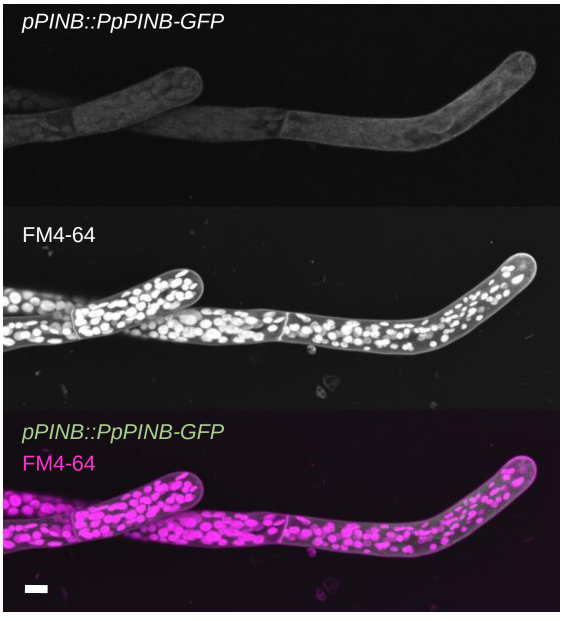

Supplemental figure 5. PpPINB is evenly distributed on the plasma membrane with a high cytosolic signal in moss protonema cells.

PpPINB-GFP is driven by its endogenous promoter and stained with FM4-64 for cell outline imaging. Scale bar = 10 µm.

Supplemental figure 6. MpPINZ-GFP signal is diminished in most elongated rhizoids.

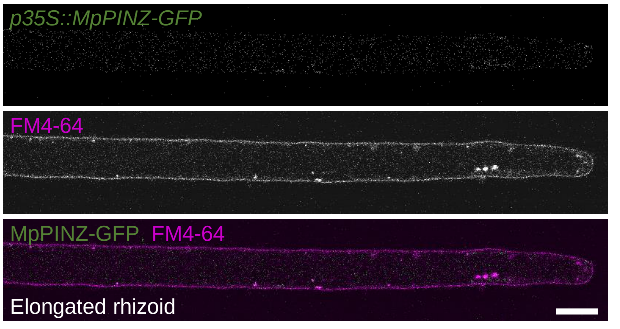

Supplemental figure 6. MpPINZ-GFP signal is diminished in most elongated rhizoids.

Elongated rhizoids were observed under the same imaging conditions as used in Figure 5B. The tissues were stained with FM4-64 for 10-30 minutes prior to imaging. Scale bar = 10 µm.

Supplemental figure 7. AtPIN1-GFP and AtPIN2-GFP show similar localization patterns under shorter induction time in moss protonema cells.

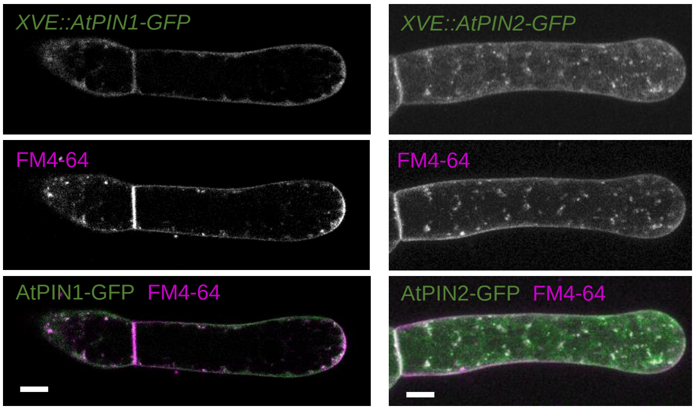

Supplemental figure 7. AtPIN1-GFP and AtPIN2-GFP show similar localization patterns under shorter induction time in moss protonema cells.

Moss tissues were cultivated in the imaging dish for 6 days and the expression of *AtPIN1-GFP* and *AtPIN2-GFP* were induced for additional 24 or 48 hours with 1 µM β-extradiol, respectively. The tissues were stained with FM4-64 for 10-30 minutes prior to imaging. Scale bar = 10 µm.

Supplemental figure 8. Arabidopsis AtPIN1 is not polarized in Marchantia.

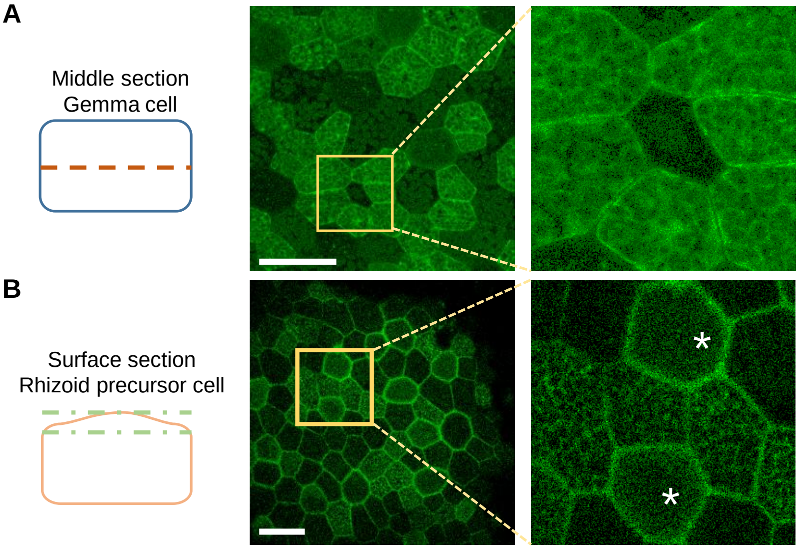

Supplemental figure 8. Arabidopsis AtPIN1 is not polarized in Marchantia.

The localization of AtPIN1-GFP in *M. polymerpha* gemma epidermal cells and protrusion site of initial rhizoids (asterisks). Imaging sections were obtained as illustrated. The AtPIN1-GFP is localized at the plasma membrane and in the cytoplasm of all examined tissues with no apparent puncta signal. Scale bar = 50 mm.

Supplemental figure 9. *MpPINZ-GFP* and *AtPIN1-GFP* driven by MpPINZ endogenous promoter show similar localization patterns in Marchantia.

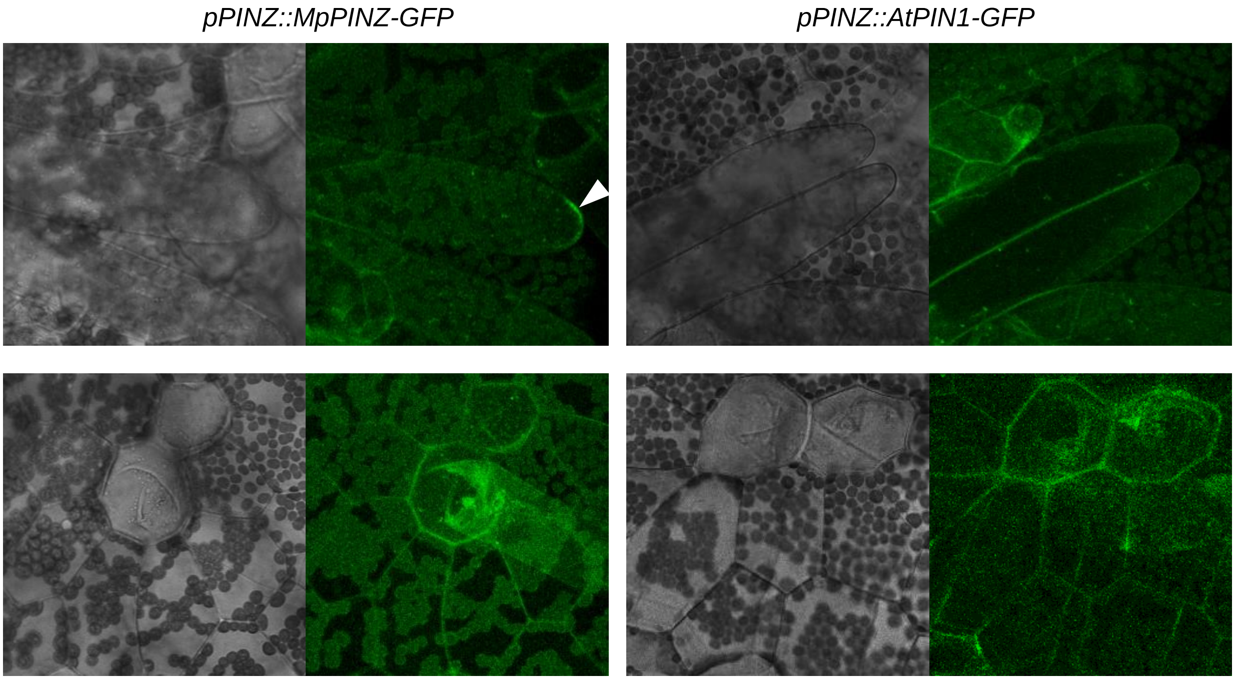

Supplemental figure 9. *MpPINZ-GFP* and *AtPIN1-GFP* driven by *MpPINZ* endogenous promoter show similar localization patterns in Marchantia.

The localization of MpPINZ-GFP and AtPIN1-GFP driven by MpPINZ endogenous promoter. MpPINZ-GFP is polarized at the tip of young rhizoids (white arrowhead), while AtPIN1-GFP is evenly distributed on the PM. Both MpPINZ-GFP and AtPIN1-GFP show PM localization with no polarity in gemma epidermal cells.

References:

1. **Ung KL, Winkler M, Schulz L, Kolb M, Janacek DP, Dedic E, Stokes DL, Hammes UZ, Pedersen BP. 2022.** Structures and mechanism of the plant PIN-FORMED auxin transporter. Nature 609(7927): 605-610.
2. **Viaene T, Landberg K, Thelander M, Medvecka E, Pederson E, Feraru E, Cooper ED, Karimi M, Delwiche CF, Ljung K, et al. 2014.** Directional auxin transport mechanisms in early diverging land plants. Curr Biol 24(23): 2786-2791.
3. **Yang Z, Xia J, Hong J, Zhang C, Wei H, Ying W, Sun C, Sun L, Mao Y, Gao Y, et al. 2022.** Structural insights into auxin recognition and efflux by Arabidopsis PIN1. Nature 609(7927): 611-615.

Supplemental Table 1. Materials used and generated in this study.

Organism	Strain	In use	Source
Physcomitrelium patens	pPINA::PpPINA-GFP	Figure 4, 6, Supplemental Figure 4	Previous study (Viaene et al., 2014)
	pPINB::PpPINB-GFP	Supplemental Figure 5	Previous study (Viaene et al., 2014)
	XVE::PpPINA-GFP	Figure 3, 5	This study
	XVE::AtPIN1-GFP	Figure 3, 5	This study
	XVE::AtPIN2-GFP	Figure 3, Supplemental Figure 7	This study
	XVE::MpPINZ-GFP	Figure 3	This study
Marchantia polymorpha	p35S::MpPINZ-GFP	Figure 3, 4, 5, 6	This study
	p35S::AtPIN1-GFP	Figure 3, 5, Supplemental Figure 8	This study
	p35S::AtPIN2-GFP	Figure 3	This study
	pPINZ::MpPINZ-GFP	Supplemental Figure 9	This study
	pPINZ::AtPIN1-GFP	Supplemental Figure 9	This study
Arabidopsis thaliana	pPIN2::AtPIN2-GFP	Figure 4, 5, 6	Previous study (Zhang et al., 2019)
	pPIN2::PpPINA-GFP	Figure 5	Previous study (Zhang et al., 2019)
	pPIN2::MpPINZ-GFP	Figure 5	Previous study (Zhang et al., 2019)

Supplemental Table 2. Plasmid and primer list used in this study.

Plasmids	Name	Description	Purpose
1	pPGX8	inducible XVE promoter, PIG locus	To generate inducible overexpression line in moss
2	pMpGWB102	Binary vector with virus 35S promoter	To generate overexpression line in Marchantia
**Primers**	**Name**	**Sequence**	**Purpose**
1	AtPIN1_TOPO_F	CACC ATGATTACGGCGGCGGACTTC	TOPO cloning
2	AtPIN1cds_R	TCATAGACCCAAGAGAATGTAGTAGAG	TOPO cloning, genotyping PCR
3	AtPIN2_TOPO_F	CACC ATGATCACCGGCAAAGACATGTAC	TOPO cloning
4	AtPIN2cds_R	TTAAAGCCCCAAAAGAACGTAG	TOPO cloning, genotyping PCR
5	PINAcds_R	TCACAGACCAAGTAATATGTAG	genotyping PCR
6	PINZcds_R	TTACAGGCCCAAAAGTACGTAG	genotyping PCR
7	PIGbR_F	GAGTCATCAAATTGAAGTACAAGTAGG	genotyping PCR
8	GFP_F	GCAAGGGCGAGGAGCTGTTC	genotyping PCR
9	GFP_R	GTACAGCTCGTCCATGCCGAG	genotyping PCR
