# Supplementary material for: Divergence of trafficking and polarization mechanisms for PIN auxin transporters during land plant evolution
Source: Plant Commun. 2023 Jul 31;5(1):100669. doi: 10.1016/j.xplc.2023.100669 (PMC10811345; doi:10.1016/j.xplc.2023.100669)
Supplement: Document S2. Article plus supplemental information [file mmc3.pdf]

Plant Communications
Research article

CellPress
Partner Journal

Divergence of trafficking and polarization mechanisms for PIN auxin transporters during land plant evolution

Han Tang[1,5,6], Kuan-Ju Lu[2,6], YuZhou Zhang[3], You-Liang Cheng[4], Shih-Long Tu[4] and Jiří Friml[1,*]

[1]Institute of Science and Technology Austria (ISTA), Am Campus 1, 3400 Klosterneuburg, Austria

[2]Graduate Institute of Biotechnology, National Chung Hsing University, No. 145, Xingda Rd., South Dist., Taichung 40227, Taiwan, R.O.C

[3]College of Life Sciences, Northwest A&F University, Shaanxi, Yangling, China

[4]Institute of Plant and Microbial Biology, Academia Sinica, 128 Sec. 2, Academia Rd, Nankang, Taipei 11529, Taiwan, R.O.C

[5]Present address: Graduate Institute of Biochemistry, National Chung Hsing University, No. 145, Xingda Rd., South Dist., Taichung 40227, Taiwan, R.O.C

[6]These authors contributed equally to this article.

***Correspondence: Jiří Friml (jiri.friml@ist.ac.at)**

https://doi.org/10.1016/j.xplc.2023.100669

ABSTRACT

The phytohormone auxin, and its directional transport through tissues, plays a fundamental role in the development of higher plants. This polar auxin transport predominantly relies on PIN-FORMED (PIN) auxin exporters. Hence, PIN polarization is crucial for development, but its evolution during the rise of morphological complexity in land plants remains unclear. Here, we performed a cross-species investigation by observing the trafficking and localization of endogenous and exogenous PINs in two bryophytes, *Physcomitrium patens* and *Marchantia polymorpha*, and in the flowering plant *Arabidopsis thaliana*. We confirmed that the GFP fusion did not compromise the auxin export function of all examined PINs by using a radioactive auxin export assay and by observing the phenotypic changes in transgenic bryophytes. Endogenous PINs polarize to filamentous apices, while exogenous *Arabidopsis* PINs distribute symmetrically on the membrane in both bryophytes. In the *Arabidopsis* root epidermis, bryophytic PINs have no defined polarity. Pharmacological interference revealed a strong cytoskeletal dependence of bryophytic but not *Arabidopsis* PIN polarization. The divergence of PIN polarization and trafficking is also observed within the bryophyte clade and between tissues of individual species. These results collectively reveal the divergence of PIN trafficking and polarity mechanisms throughout land plant evolution and the co-evolution of PIN sequence-based and cell-based polarity mechanisms.

Keywords:: *Arabidopsis*, evolution, *Marchantia*, *Physcomitrium*, PINs, polarization, protein trafficking

Tang H., Lu K.-J., Zhang Y., Cheng Y.-L., Tu S.-L., and Friml J. (2024). Divergence of trafficking and polarization mechanisms for PIN auxin transporters during land plant evolution. Plant Comm. **5**, 100669.

INTRODUCTION

Auxin is a crucial regulator of polarity and morphogenesis in land plants (Mockaitis and Estelle, 2008; Smit and Weijers, 2015; Kato et al., 2018; Leyser, 2018; Yu et al., 2022). The auxin gradients and local maxima within tissues coordinate a broad spectrum of plant development, ranging from embryogenesis to organ formation and tropisms (Vanneste and Friml, 2009; Friml, 2021). The establishment of the auxin gradient relies predominantly on directional auxin transport driven by the PIN-FORMED (PIN) efflux carriers. In different tissue types, specific PINs are polarized at different plasma membrane (PM) domains, directly driving the directionality of auxin flow (Adamowski and Friml, 2015). Therefore, given the essential impact of auxin flow in various developmental processes, the function and polarization of PIN proteins are crucial for maintaining the correct pattern of plant growth and patterning (Sauer and Kleine-Vehn, 2019).

PINs are found in all land plants and can be traced back to charophytic green algae (Viaene et al., 2013; Skokan et al., 2019). Functional conservation of PINs in auxin transport has also

Published by the Plant Communications Shanghai Editorial Office in association with Cell Press, an imprint of Elsevier Inc., on behalf of CSPB and CEMPS, CAS.

 © 2023 The Authors.
This is an open access article under the CC BY license (http://creativecommons.org/licenses/by/4.0/).

been demonstrated by exogenously expressing *PINs* from the green algae *Klebsormidium flaccidum*, the moss *Physcomitrium patens*, and the angiosperm *Arabidopsis thaliana* in transgenic plants and in heterologous systems (Zourelidou et al., 2014; Skokan et al., 2019). Additionally, when exogenous *PINs* from charophytes or *Arabidopsis* are overexpressed in *P. patens*, the transgenic plants show growth inhibition that resembles auxin deprivation (Viaene et al., 2014; Lavy et al., 2016; Tao and Estelle, 2018; Skokan et al., 2019). These observations support the hypothesis that PIN-mediated polar auxin transport has been governing plant development since the emergence of land plants.

PIN polarity regulation has been investigated extensively in the angiosperm *Arabidopsis*. Canonical PINs contain a long central hydrophilic loop (HL) between two transmembrane domains and are delivered to the PM via the endoplasmic reticulum-Golgi apparatus vesicle trafficking pathway. Depolymerization of actin filaments induces accumulation of PIN-labeled small intracellular puncta near the PM but with no apparent PIN polarity defect (Geldner et al., 2001; Glanc et al., 2018; 2019). Microtubules are involved in the cytokinetic trafficking of PINs but are not required for polarity establishment or maintenance at the PM of non-dividing cells (Geldner et al., 2001; Kleine-Vehn et al., 2008b; Glanc et al., 2019). Notably, disruption of both cytoskeletal networks delays, but does not abolish, AtPIN2 polarization in *Arabidopsis* epidermal cells (Glanc et al., 2019). This suggests that the cytoskeletal networks participate in but are not strictly essential for PIN polar trafficking, whereas other mechanisms contribute to PIN polar localization. PINs are known to undergo constitutive cycles of endocytosis and recycling, which is modulated by auxin itself (Narasimhan et al., 2020, 2021). This process is essential for their polar distribution (Kleine-Vehn et al., 2011; Doyle et al., 2015).

Phosphorylation of specific sites within the HL region is a critical determinant for the apical-basal polarization pattern of PINs in *Arabidopsis* epidermal cells. A serine/threonine kinase, PINOID, phosphorylates specific sites on AtPIN2 and leads to its apical localization (Friml et al., 2004). In contrast, when phosphatase 2A dephosphorylates AtPIN2 it counteracts PINOID-dependent phosphorylation and guides the delivery of AtPIN2 to the basal domain of epidermal cells (Michniewicz et al., 2007). The phosphorylation sites targeted by different kinase families are crucial for polar localization and PIN function, and most sites are highly conserved within canonical *Arabidopsis* PINs (Zwiewka et al., 2019). Because PINs are present in all land plants, one can hypothesize that phosphorylation-based polarity regulation may have been established since the emergence of early land plants. However, it has never been demonstrated that these phosphorylation sites are evolutionarily conserved in early land plants.

PIN polarization has been observed in the moss *P. patens*, which grows as filamentous protonemata. PpPINA-GFP exhibits polar localization at the tip of protonema cells, but polar localization of PpPINA-GFP is not always conserved in other species (Bennett et al., 2014; Viaene et al., 2014). When *PpPINA-GFP* is expressed in *Arabidopsis* root epidermal cells, where AtPIN2-GFP exhibits clear apical localization, PpPINA-GFP localizes to basal and apical sites (Zhang et al., 2019). Furthermore, PINs from the liverwort *Marchantia polymorpha* and from the green alga *K. flaccidum* are also mislocalized in root epidermal cells in *Arabidopsis* (Zhang et al., 2019). This distinctive PIN localization pattern in different species suggests that mechanisms of PIN trafficking and polarization may have diversified after the emergence of land plants. Despite the profound significance of PIN polarization, and the resulting directional auxin transport for land plant development, PIN trafficking and polarization mechanisms are mainly derived from the angiosperm model *A. thaliana*.

In this study, we investigated PIN trafficking/polarization mechanisms from an evolutionary perspective. We show that canonical PINs from the bryophytes *P. patens* and *M. polymorpha* and the land plant *Arabidopsis* exhibit high conservation in their transmembrane domains and phosphorylation sites. Endogenous PIN-GFP shows different localization patterns in various developmental contexts, suggesting tissue-specific PIN polarization mechanisms. A cross-species investigation revealed that exogenous PINs can traffic to the PM but fail to enrich at the polar domains, unveiling species-specific mechanisms for PIN polarization. This notion was verified by a different dependency of the cytoskeleton for polarization of *Arabidopsis* PINs and bryophytic PINs. Overall, our results highlight that PIN trafficking and polarization mechanisms underwent complex evolution during the gradual rise of morphological complexity in land plants.

RESULTS

Phosphorylation sites are highly conserved between bryophytic and *Arabidopsis* PINs

We performed a phylogenetic analysis to better understand the extent of conservation between bryophytic and *Arabidopsis PINs*. The coding sequences of the single canonical PIN *MpPINZ* from *M. polymorpha*, three canonical PINs (*PpPINA–PpPINC*) from *P. patens*, and five canonical PINs (*AtPIN1–AtPIN4* and *AtPIN7*) from *Arabidopsis* were aligned using MEGA X (Kumar et al., 2018). Bryophytic PINs cluster together, and *MpPINZ* is more closely related to *Arabidopsis PINs* than *PpPINs* (Figure 1A). *Arabidopsis* AtPIN1 and AtPIN2 exhibit a polar localization pattern at the PM that plays a crucial role in embryogenesis, organ formation, and tropic growth (Krecek et al., 2009; Omelyanchuk et al., 2016). The phylogenetic tree shows that AtPIN1 and AtPIN2 are equally close to bryophytic PINs, and the polarized localization pattern of AtPIN2 has been studied extensively in roots (Abas et al., 2006; Kleine-Vehn et al., 2008a; Glanc et al., 2018). Therefore, we used AtPIN2 as our reference for further alignment analyses. The identity index of coding amino acid sequences showed that full-length AtPIN2 shares around 50% identity with each bryophytic PIN (Figure 1B). We suspected that central HLs would be more divergent since the transmembrane domains show high similarity between all examined PINs. Surprisingly, the identity index for the HL region of AtPIN2 shared over 40% identity with the HL region of MpPINZ (45%), PpPINA (42%), and PpPINB (43%) (Figure 1B). The identity index of full-length AtPIN1 and HLs showed similar results as AtPIN2 (Supplemental Figure 1).

The overview of coding sequences for all examined PINs revealed highly conserved transmembrane domains at the N-terminus and C-terminus that were connected by a less conserved HL region

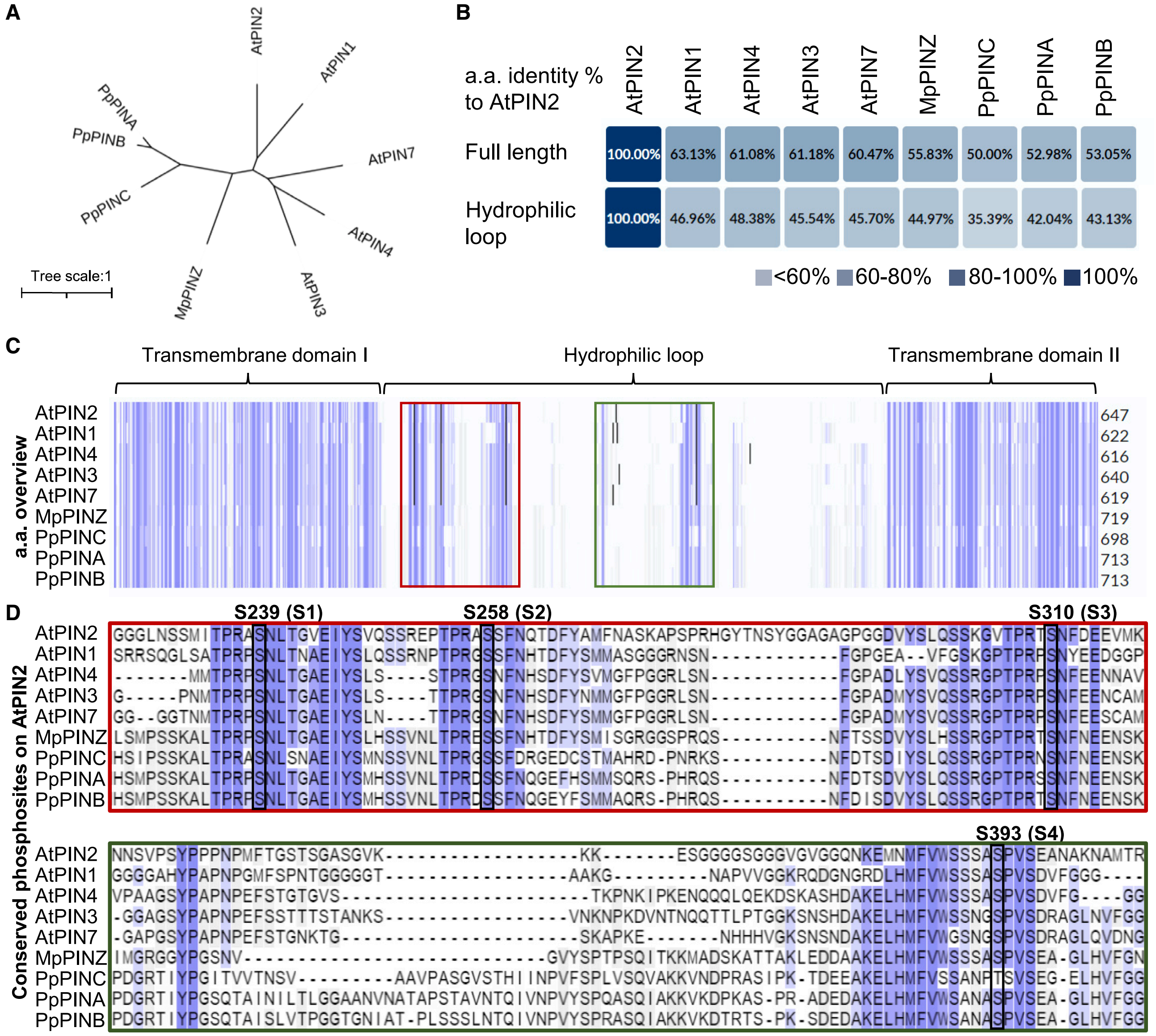

Figure 1. Phosphorylation sites are highly conserved between bryophytic and *Arabidopsis* PINs.

(A) Phylogenetic analysis of canonical PINs from the early-divergent plants *P. patens* (*Pp*) and *M. polymorpha* (*Mp*) and the representative angiosperm *A. thaliana* (*At*). The unrooted tree shows the relationships between different PINs in two representative bryophytes and *Arabidopsis*. The scale bar represents the number of changes per site.

(B) Identity indexes of all PINs compared with AtPIN2 with full-length or only the HL region of coding amino acid sequences. Identity indexes of all PINs compared with AtPIN1 are shown in Supplemental Figure 1.

(C) Alignment of PIN amino acid sequences. Identical amino acids are highlighted with blue columns. The red and green boxes highlight the HL regions bearing conserved phosphorylation sites, which are enlarged in **(D)**.

(D) Four conserved phosphorylation sites, labeled S1-S4, were verified in previous AtPIN2 studies and are depicted with black frames. Note that S1-S4 are conserved in every examined PIN.

(Figure 1C). Because polarization of AtPIN2 is tightly associated with the phosphorylation status of the HL region, we used AtPIN2 as a reference to search for and highlight these experimentally identified phosphorylation sites (Sukumar et al., 2009; Zhang et al., 2010; Barbosa et al., 2018). Compared with transmembrane domains, despite relatively lower conservation in their HLs, the four identified phosphorylation sites are fully conserved between *Arabidopsis* PINs and bryophytic PINs (Figure 1D), which suggests that PIN phosphorylation might be evolutionarily conserved to regulate the intracellular localization of PIN proteins.

The HL regions in AtPIN2, PpPINA, and MpPINZ are less conserved

We used Alphafold2 to predict the structures of *Arabidopsis and bryophytic PINs and performed structural* alignments to assess their overall structural conservation. These structures closely

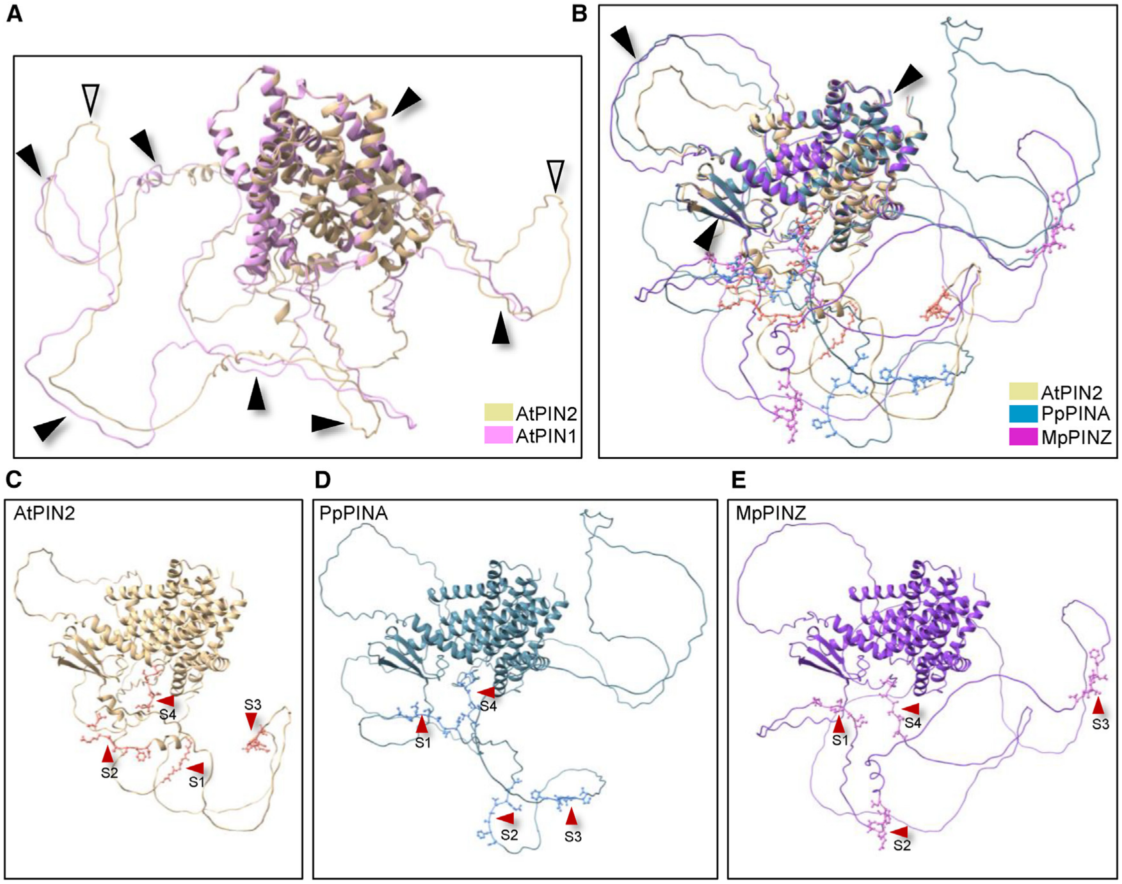

Figure 2. The HL regions in AtPIN2, PpPINA, and MpPINZ are less conserved.

(A) Structural alignment of AtPIN1 and AtPIN2. The protein structures were predicted using Alphafold2 and aligned using ChimeraX. The structurally conserved regions are indicated by black arrowheads, and the non-conserved regions are indicated by white arrowheads.

(B) Structural alignment of AtPIN2 with PpPINA and MpPINZ. Only the transmembrane domains and a single loop align with each other, while the majority of HL regions are not conserved. The four conserved phosphorylation sites are labeled with atomic details in ball-and-stick style.

(C–E) Individual protein structures retrieved from **(B)**. The four conserved phosphorylation sites are indicated by red arrowheads.

resembled the crystal structures of AtPIN1 and AtPIN8 (Supplemental Figure 2A and 2B; Jumper et al., 2021; Ung et al., 2022; Yang et al., 2022), which suggested that the Alphafold2 structure predictions were reliable. We compared these structures to the well-characterized and polarized AtPIN1 and AtPIN2 using Alphafold2 for structure prediction and ChimeraX for structure alignments (Jumper et al., 2021; Pettersen et al., 2021). The structures of the transmembrane domains were highly conserved, and the HL regions shared similar folds (Figure 2A, black arrowheads) except for two additional loops in AtPIN2 (Figure 2A, white arrowheads). We next aligned the structure of AtPIN2, PpPINA, and MpPINZ. The predicted transmembrane domains of these three PINs had very high confidence scores in Alphafold2 and were highly conserved with nearly perfect alignment. However, the HL regions had very low confidence scores and were less conserved with only one loop sharing partial similarity (Figure 2B and Supplemental Figure 2C, black arrowheads). Conserved phosphorylation sites were observed by rotating the aligned protein structures presented in Figure 2C–2E. In general, bryophytic PINs possess looser and larger loops compared with AtPIN2 (Figure 2B–2E). The predicted structures are shown individually with the annotated phosphorylation sites that are indicated in Figure 1D (Figure 2C–2E). The structural conservation of transmembrane domains implies that bryophytic PINs may traffic to the PM as *Arabidopsis* PINs do, whereas the loose loops with conserved phosphorylation sites suggest that their polarization pattern may be different.

GFP-fused PIN proteins possess auxin export activity

The sequence and structural analyses of *Arabidopsis* and bryophytic PINs revealed high conservation in sequence, phosphorylation sites, and structure of the transmembrane domains. We next investigated whether bryophytic PINs are delivered to the PM with polar domain enrichment as *Arabidopsis* PINs. We determined the localization pattern of the PIN proteins by fusing *GFP* to *AtPIN1*, *AtPIN2*, *PpPINA*, and *MpPINZ* as shown in Figure 3A (Zhang et al., 2019). Auxin export mediated by these PIN-GFP fusions was assessed by subcloning each fusion gene into a moss vector and driving expression with an inducible *XVE* promoter (Kubo et al., 2013). We generated transgenic moss plants expressing single *XVE::PIN-GFP* transgenes and verified their genotypes (Supplemental Figure 3). We used these transgenic lines to perform the auxin export assay. In brief, overexpression of *PIN-GFPs* was

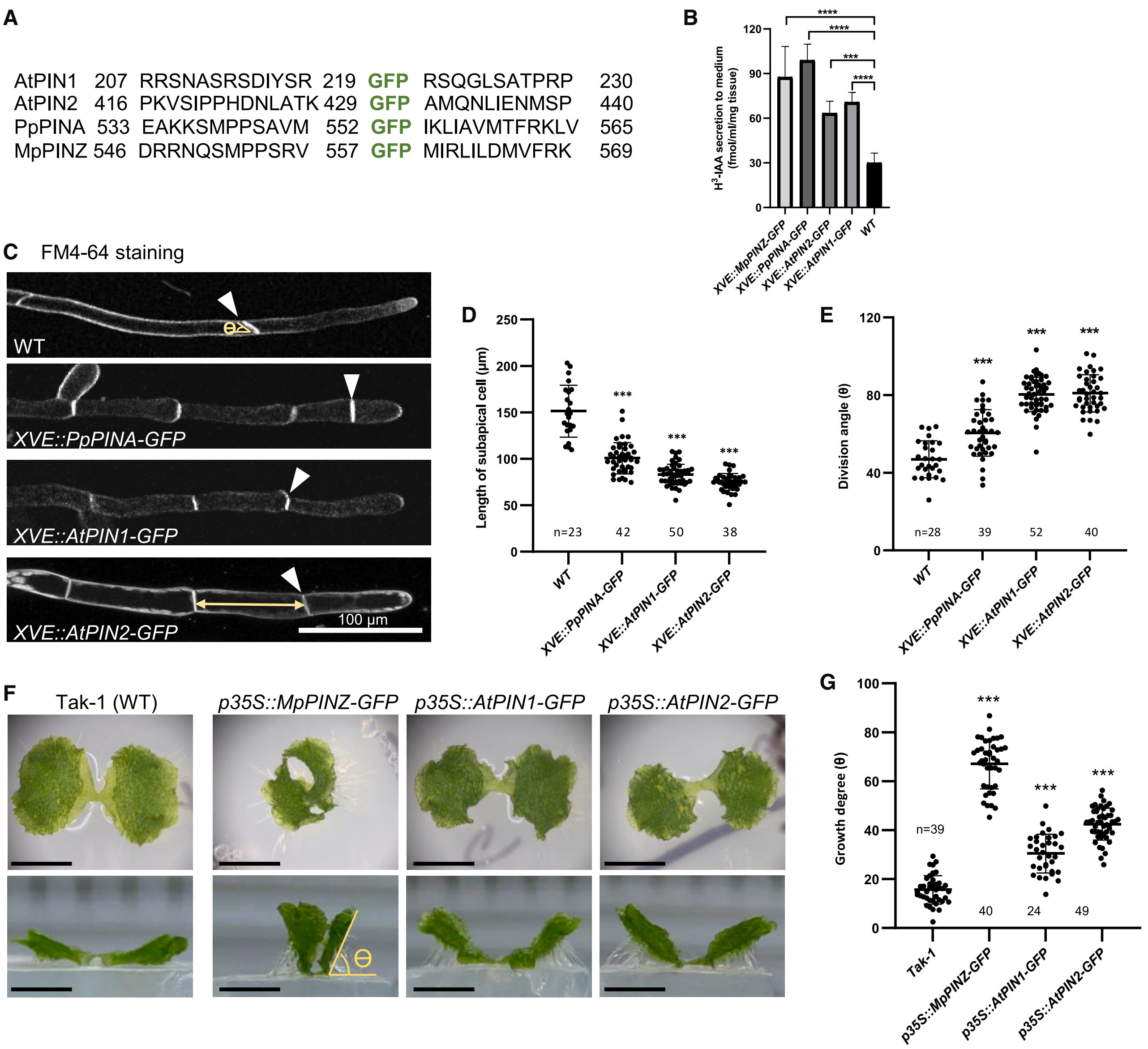

Figure 3. GFP-fused PIN proteins possess auxin export activity.

(A) The insertion site of GFP in the indicated PIN proteins. Numbers represent the amino acid position in the respective proteins.

(B) The auxin export assay with *P. patens* transgenic plants. Fresh tissues were cultured in liquid medium supplied with 1μM β-estradiol to induce XVE overexpression. Tissues were then incubated with radioactive H^3-IAA followed by washing. Radioactive H^3-IAA exported into the new culture medium was measured using a scintillation detector after one day. Wild-type moss plants treated with the same conditions were used as a control. Ten to fifteen 10-day-old moss colonies were used for one measurement, and the graph shows the mean ± SD from four independent experiments.

(C) Representative protonema cells of the indicated genetic background. The cell outline was stained with FM4-64. White arrowheads indicate the first cell division plane, and the yellow double arrow indicates the length of the subapical cell. Scale bar, 100 μm.

(D and E) Quantification of the subapical cell length and division angle (Θ) of the indicated lines. Bold horizontal lines indicate the median, and whiskers indicate the first and third quartile. ***P < 0.001, Student's t-test.

(F) Top (top panels) and side (bottom panels) views of the indicated *M. polymorpha* lines. Takaragaike-1 (WT) expands its thallus horizontally on the agar surface. Overexpression of *MpPINZ-*, *AtPIN1-*, and *AtPIN2-GFP* showed vertical thallus growth, which generated a large angle between the lower surface of the thallus and the surface of the agar (Θ). Scale bar, 0.5 cm.

(G) Quantification of the thallus growth angle (Θ) shown in **(A)**. Bold horizontal lines indicate the median, and whiskers indicate the first and third quartile. ***P < 0.001, Student's t-test.

induced by β-estradiol for 3 days, followed by radioactive auxin H^3-IAA treatment for 24 h. The radioactive tissues were then washed twice and incubated in fresh growth medium for another 24 h. The culture medium was collected for H^3 scintillation detection (Lewis and Muday, 2009). Wild-type (WT) moss plants were used as an internal control to show basal exportation of H^3-IAA by endogenous PpPINs. In comparison with the WT, all examined PIN-GFP plants showed a higher amount of radioactive auxin in the culture medium, indicating their auxin export activity (Figure 3B).

The function of PIN-GFPs was also confirmed by the growth changes caused by *PIN-GFP* overexpression in *P. patens* and *M. polymorpha*. During early development, *P. patens* gradually transits its filamentous protonemata from thicker/shorter chloronema cells with perpendicular division planes to thinner/longer caulonema cells with oblique division planes (Rensing et al., 2020). *PpPINA*, *PpPINB*, and *PpPIND* overexpression has previously been shown to inhibit protonema transition (Viaene et al., 2014). Here we showed that overexpression of *PpPINA*-, *AtPIN1*-, and *AtPIN2-GFP* led to similar defects in this chloronema-caulonema transition, with a shorter length of the subapical cell and a larger division angle (Figure 3C–3E). Wild-type *M. polymorpha* has prostrate thalli. However, with overexpression of either *MpPINZ-GFP* or *AtPIN-GFPs* (genotypes are confirmed in Supplemental Figure 3), the thallus grew more vertically, as apparent from the side view (Figure 3F), phenocopying the auxin-deficient phenotype (Kato et al., 2017). The angles between the thallus and the horizontal agar were measured to quantify vertical growth (Figure 3G). Overexpression of *MpPINZ-GFP* caused the most striking phenotype, but overexpression of *AtPIN-GFPs* also resulted in vertical growth that showed significant differences from the WT. Our results show that all PIN-GFPs can export auxin at least in the moss system and that overexpression of *PIN-GFPs* causes phenotypic changes in *P. patens* and *M. polymorpha*.

Endogenous PINs exhibit different localization patterns in different tissue types

We utilized a stable moss transgenic line expressing the *PpPINA genomic DNA-GFP* fusion under its native promoter (*pPINA::PpPINA-GFP*) to observe PpPINA-GFP localization in *P. patens.* The moss *P. patens* has a filamentous protonema stage and a leafy gametophore stage in its life cycle. The initial protonema cell was regenerated from a detached leaf, and the elongated protonema was imaged in a six-day-old moss colony. PpPINA-GFP localized at the PM of the protonema tip with a clear polarity in both initial and elongated protonemata (Figure 4A). This polarization pattern also appeared in chloronema and caulonema cells. To determine if polar localization of PpPINA-GFP occurs in complex tissues composed of multiple cell layers, we observed its localization in gametophytic leaves. Near the tip of gametophytic leaves, PpPINA-GFP showed clear basal-apical polarization along the leaf axis with notable corner enrichment (Figure 4A) and was evenly distributed on the PM near the base of the leaves (Supplemental Figure 4).

We extended our analysis to *PpPINB* fused to GFP and driven by its endogenous promoter. Notably, PpPINB-GFP did not show visible polarity at the tip of protonema but had a more even PM distribution with an increased intracellular signal (Supplemental Figure 5). This distribution pattern of PpPINB-GFP differs from a previous study and could be due to reduced expression of our *PpPINB-GFP* construct since it was driven by the native *PpPINB* promoter as opposed to overexpression in the previous study (Viaene et al., 2014). The differences between the localization of PpPINA-GFP and PpPINB-GFP suggest that these PINs may be recruited by distinct polarization pathways or have different regulation in the same cell.

The divergence of PpPINA-GFP polarity in filamentous protonema cells and in gametophytic leaves made us wonder whether this tissue-specific polarization of PINs is conserved in other bryophytes. To determine this, we examined the bryophyte and liverwort *M. polymorpha*, which produces gemmae as the asexual reproductive progenies that consist of multiple cell layers. After water imbibition, single-cell rhizoids emerge from the large rhizoid precursor cells on the epidermis of a gemma (Shimamura, 2016). We generated a *p35S::MpPINZ-GFP* transgenic line to determine the subcellular localization of the sole canonical PIN in *M. polymorpha*. Interestingly, MpPINZ-GFP localized on the PM with small intracellular puncta and no apparent polarity in all gemma epidermal cells (Figure 4B, right panel). However, when gemmae were stimulated to grow rhizoids, MpPINZ-GFP accumulated at the protrusion site of emerging rhizoids in the rhizoid precursor cells (Figure 4B, yellow arrowheads). MpPINZ-GFP accumulation was lost in most young and elongated rhizoids shortly after their emergence. However, around 5%–10% of the observed young rhizoids had weak MpPINZ-GFP accumulation at their tips and a polar localization pattern (Figure 4B and Supplemental Figure 6).

The localization pattern of PpPINA- and MpPINZ-GFP in filamentous and complex tissues suggests distinct polarity recognition mechanisms for bryophytic PINs in different tissue types. To examine whether *Arabidopsis* PINs show similar polar patterns as bryophytic PINs in different types of tissues, we expressed *pPIN2::AtPIN2-GFP* in filamentous root hairs and complex epidermal cells. AtPIN2-GFP exhibited apical localization in epidermal cells, which is consistent with previous observations (Figure 4C; Zhang et al., 2019). We next observed AtPIN2-GFP in initial and elongated root hairs to see if localization was consistent with the polar localization of bryophytic PINs at the tip of filamentous tissues. AtPIN2-GFP exhibited polar localization at the tip of initial root hairs (Figure 4C). However, the polarity of AtPIN2-GFP signals diminished in elongated hairs (Figure 4C). The different polarity patterns of PINs in different types of tissue support the notion that regulation of PIN polarization is specialized in different cellular profiles and developmental contexts.

Exogenous PINs are localized to the PM with no defined polarity

Because bryophytic and *Arabidopsis* PINs demonstrated tissue- and development-specific polarity regulation, we wondered if conserved phosphorylation sites in the HL region are sufficient to drive polarization of exogenous PINs in other species. We utilized the same moss *XVE::PIN-GFP* transgenic lines generated for the auxin export assay to compare *PpPINA-, AtPIN1-, and AtPIN2-GFP.* In protonemata, AtPIN1-GFP was evenly distributed on the PM and cell division plane with no polarity at the tips and displayed numerous small intracellular puncta, whereas PpPINA-GFP had the same polarization pattern as when it was driven by its endogenous promoter (Figures 4A, 5A, and 5D). The signal from AtPIN2-GFP under the same induction condition had a much lower intensity but resembled AtPIN1-GFP localization patterns, so we used AtPIN1-GFP localization for our representative images (Supplemental Figure 7). Short-term weak induction of *XVE::AtPIN1-GFP* was performed, and it showed no difference in localization, which verified that the localization patterns of AtPIN1-GFP were not caused by overexpression (Supplemental Figure 7; Supplemental Video 1).

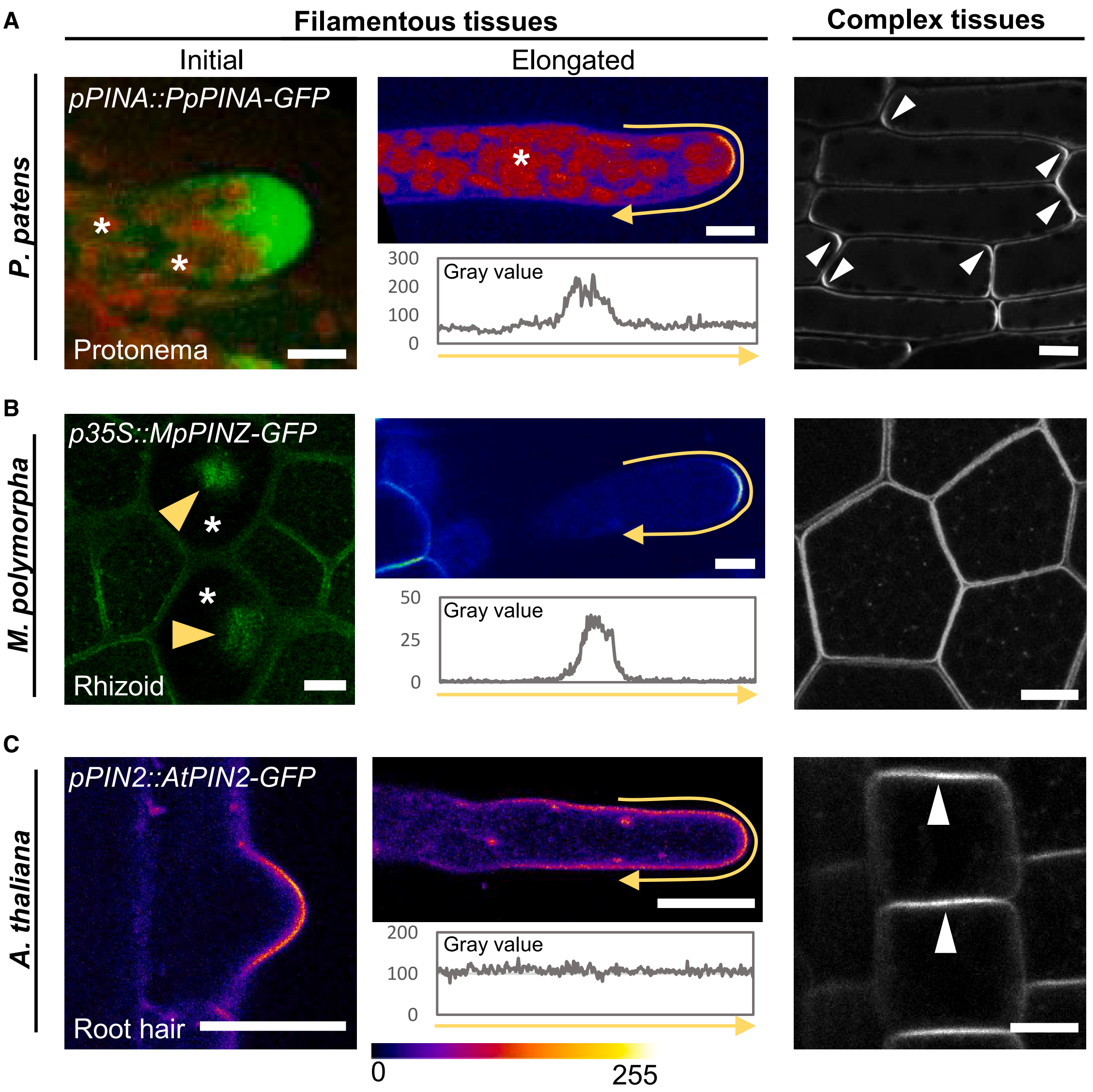

Figure 4. Endogenous PINs present different localization patterns in different tissues.

(A) The localization of PpPINA-GFP in tissues with different complexities. PpPINA-GFP is polarized to the tip of initial and elongated protonema cells. The initial protonema cell is regenerated from a detached leaf, and the representative image shows the maximum projection with a 5-μm-thick Z section. Autofluorescence from the chloroplasts is indicated by asterisks. In elongated protonema cells, the polarity of PpPINA-GFP is plotted using an intensity measurement along the PM, as represented by the yellow arrows. The same measurement is applied to **(B)** and **(C)**. In complex tissues composed of multiple cell layers (e.g. young leaves in *P. patens*), PpPINA-GFP is polarized at apical and basal domains (white arrowheads). Scale bars, 10 μm.

(B) The localization of MpPINZ-GFP in emerging rhizoids, young rhizoids, and gemma epidermal cells. A representative image with a 5-μm-thick Z section at the gemma surface shows the accumulation of MpPINZ-GFP at the tips of emerging rhizoids, as indicated by yellow arrowheads in rhizoid precursor cells (asterisks). MpPINZ-GFP is polarized at the tip of young rhizoids (center). A middle section image shows the even distribution of MpPINZ-GFP on the PM of gemmae composed of multiple cell layers. Scale bars, 10 μm.

(C) AtPIN2-GFP shows a polarized signal at the tip of the initial root hair, but the polarized signal is not observed in elongated root hairs. All imaging details are described in the Methods. The pixel values ranging from 0-255 are represented by the rainbow color. In *Arabidopsis* epidermal cells, AtPIN2-GFP shows apical polarization, as indicated by white arrowheads. Scale bars, 10 μm for root hairs and 1 cm for epidermis.

To investigate whether these features are conserved in bryophytes, we expressed *AtPIN1-GFP* using a *35S* promoter in *M. polymorpha*. AtPIN1-GFP exhibited non-polar PM localization with high cytosolic signals in gemma epidermal cells (Supplemental Figure 8A). No visible polar localization was observed in rhizoid precursor cells of emerging rhizoids (Supplemental Figure 8B). MpPINZ-GFP exhibited clear tip polarization in young rhizoids, whereas AtPIN1-GFP was evenly distributed on the PM with homogeneous cytosolic signals (Figure 5B and 5D). To determine if the polar localization pattern of MpPINZ-GFP and the apolar localization pattern of AtPIN1-GFP in *M. polymorpha* were due to overexpression, we expressed each gene using the endogenous *MpPINZ* promoter. The fluorescent signal in rhizoids was much weaker, but the localization patterns were consistent for both proteins with either promoter driving expression (Supplemental Figure 9).

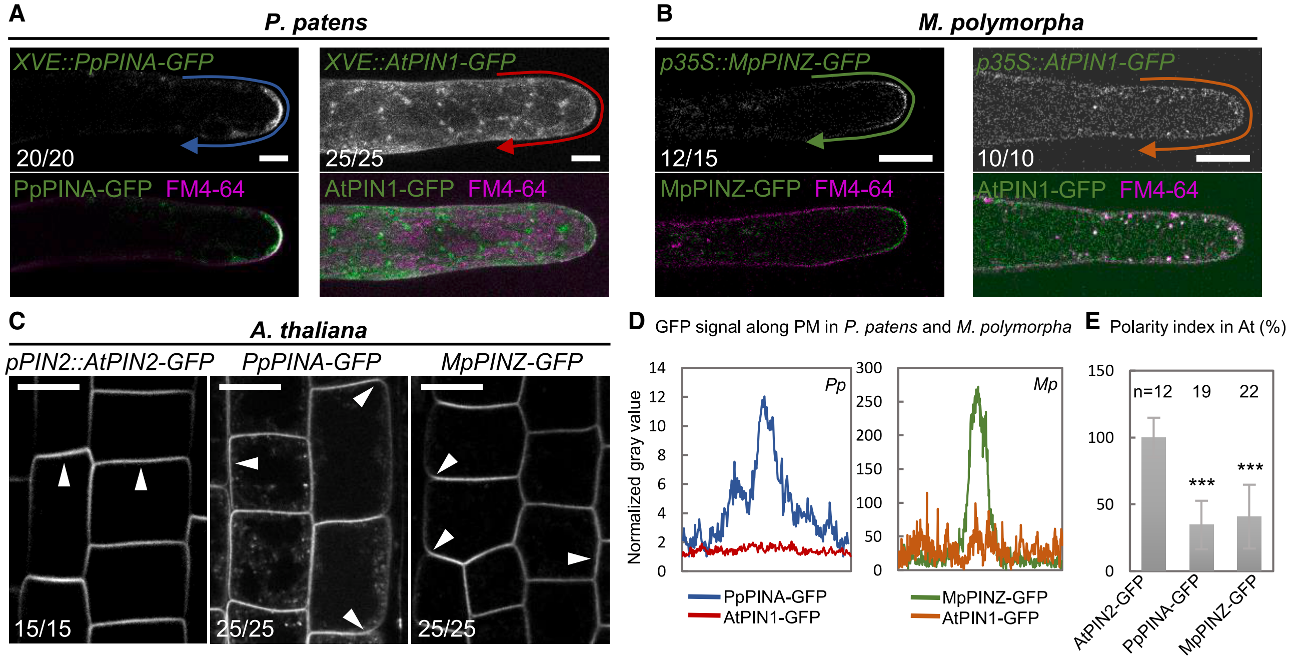

Figure 5. Exogenous PINs are PM-localized with no defined polarity.

(A) Overexpressed PpPINA-GFP is polarized to the tip of protonemata, but overexpressed AtPIN1-GFP is evenly distributed on the PM with intracellular puncta. The PM is stained with the membrane dye FM4-64. The occurrence frequency is indicated in the bottom left corner of each image. Scale bars, 10 μm.

(B) Overexpressed MpPINZ-GFP is polarized to the tip of young rhizoids, while overexpressed AtPIN1-GFP shows strong cytosolic signals and weak PM localization with no polarity. The PM is stained with the membrane dye FM4-64. Scale bars, 10 μm.

(C) AtPIN2-GFP under its native promoter localizes to the apical side of epidermal cells, but PpPINA-GFP and MpPINZ-GFP are mislocalized to the basal and lateral sites, as indicated by white arrowheads. Scale bar, 1 cm.

(D) Intensity plots of representative images for PpPINA-GFP, AtPIN1-GFP in *P. patens* protonema cells, and MpPINZ-GFP and AtPIN1-GFP in *M. polymorpha* young rhizoids.

(E) Polarity index (ratio of signal intensity at the apical PM/signal intensity at the lateral PM) for apical localization of the indicated PIN-GFP in *Arabidopsis* epidermal cells. PpPINA-GFP and MpPINZ-GFP are significantly lower on the polarity index. Shown are 12-22 cells from three roots in three independent experiments for each line. ***$P < 0.001$, Student's *t*-test.

Our results indicated that AtPIN1-GFP is delivered to the PM with no visible polarity when expressed in bryophytes. We next wanted to know if *Arabidopsis* trafficking machinery can polarize bryophytic PINs. To analyze bryophytic PINs, we observed protein localization in the epidermal cells of transgenic *Arabidopsis* lines expressing *pPIN2::AtPIN2-*, *PpPINA-*, and *MpPINZ-GFP* (Zhang et al., 2019). AtPIN2-GFPexhibited apical localization in epidermal cells, PpPINA-GFP localized at the apical and basal sides, and MpPINZ-GFP mainly localized at the basal side with some lateral localization (Figure 5C and 5E; Zhang et al., 2019). These data suggest that the *Arabidopsis* trafficking machinery drives PIN proteins to the PM through a generally conserved cellular trafficking pathway, whereas PIN polarization is specialized in different species.

Cytoskeletal networks are important for the polarization of bryophytic PINs

The diversification of PIN polarities in different plant species and tissues suggests that plant cells might utilize distinct machineries to deliver and maintain PIN proteins to the target side on the PM. Cytoskeletal networks guide directional vesicle trafficking in all eukaryotes and play critical roles in the maintenance and establishment of cell polarity in animal cells (Li and Gundersen, 2008). To verify the necessity of the cytoskeletal networks in the polarization of bryophytic PINs and AtPIN2 in their native species, we depolymerized actin filaments or microtubules by treating plant tissues with latrunculin B (LatB) or oryzalin (Ory), respectively. In *P. patens*, the disruption of actin filaments resulted in the hyperpolarization of PpPINA-GFP, which accumulated at a focal locus at the very tip of the cell (Figure 6A and 6D). Disruption of microtubules resulted in less accumulation of PpPINA-GFP at the tip of protonemata, and PpPINA-GFP appeared to be detached from the PM (Figure 6A). Changes in PpPINA-GFP localization in response to drug treatment demonstrated a requirement for cytoskeletal networks for polarization in filamentous tissues.

We used the same pharmacological interference to investigate whether MpPINZ-GFP polarization in young rhizoids of *M. polymorpha* relies on the cytoskeletal network. MpPINZ-GFP remained polarized at the tip when actin filaments were disrupted, whereas disruption of microtubules resulted in dislocation of the polarized MpPINZ-GFP (Figure 6B and 6E). These results collectively demonstrate the diversification of cytoskeletal dependency for PIN polarization within the bryophyte clade.

AtPIN2-GFP localization was monitored in the initial root hairs of *Arabidopsis* to determine if the cytoskeletal network was required

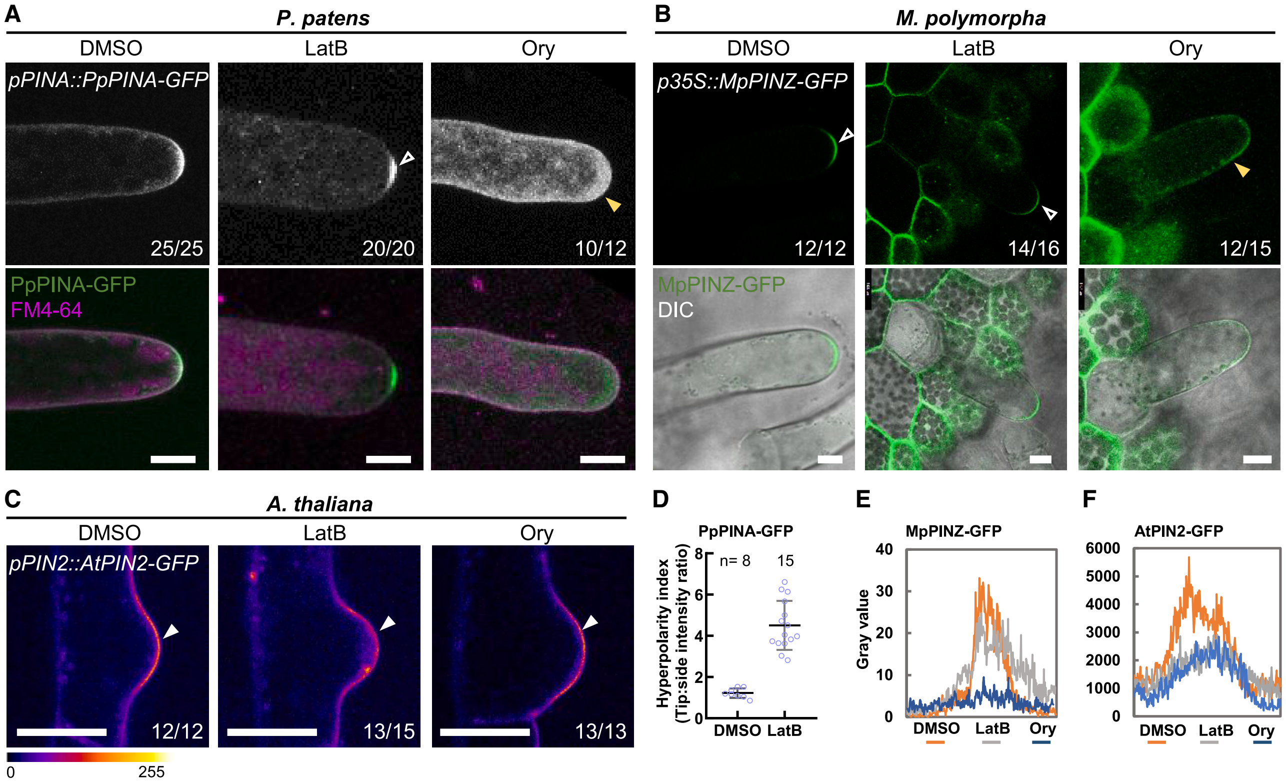

Figure 6. Cytoskeletal networks are important for the polarization of bryophytic PINs.

(A) PpPINA-GFP is polarized to the tip of protonemata, and disruption of actin filaments with latrunculin (LatB) induced its hyperpolarization (white arrowhead). Disruption of microtubules by oryzalin (Ory) disturbed the polarization of PpPINA-GFP (yellow arrowhead). The occurrence frequency is indicated in the bottom right corner of each image. For *P. patens* and *A. thaliana*, tissues were treated with 20 μM LatB or 5 μM Ory for 4 h. Scale bars, 10 μm.

(B) MpPINZ-GFP is polarized to the tip of young rhizoids (white arrowheads), and its polarization was only abolished by Ory treatment (yellow arrowhead). The gemmae were treated with 2 μM LatB or 10 μM Ory for 2 h. Scale bars, 10 μm.

(C) AtPIN2-GFP is polarized to the tip of initial root hair cells, and disruption of either actin filaments or microtubules did not change its polarization (white arrowheads) but did attenuate the peak signal at the tips, as shown in **(F)**. The pixel values ranging from 0-255 are represented by the rainbow color. Scale bars, 10 μm.

(D) The hyperpolarity index of PpPINA-GFP treated with DMSO or LatB was calculated by dividing the intensity at the very tip by the intensity at the curvature side of the tip.

(E and F) Intensity plots of representative images for MpPINZ-GFP and AtPIN2-GFP along the tips of the rhizoids in *M. polymorpha* and root hairs in *A. thaliana.*

for PIN polarization during tip cell growth. Despite attenuation of the AtPIN2-GFP signal at the tip of initial root hair cells, disruption of either actin filaments or microtubules had a minor effect on AtPIN2-GFP polarization (Figure 6C and 6F). Upon cytoskeleton disruption, apical polarization of AtPIN2-GFP in root hair cells was still evident, suggesting that, in contrast to the dependency of cytoskeletal networks for the bryophytic PINs, polarization of *Arabidopsis* PINs mainly relies on other trafficking or polarity retention mechanisms. These results reinforce the notion that mechanisms underlying PIN polarization have been diversified between bryophytes and vascular plants.

DISCUSSION

Evolution of sequence-specific determinants of PIN polarity

The flowering plant *Arabidopsis* has five PM-localized canonical PINs that exhibit different polarities in different developmental and tissue contexts. These differences in polarity help mediate directional auxin fluxes and generate asymmetric auxin distribution for a plethora of developmental processes, which ultimately shape the plant form. The PIN family originated from a single PIN auxin transporter, such as those found in simple filamentous streptophyte algae, but radiated during evolution into PINs with different expression and localization patterns that mediate diverse developmental and physiological processes (Skokan et al., 2019).

Ectopic co-expression of PINs in the same cell type can result in different polarity patterns. For example, AtPIN1 exhibits a basal pattern and AtPIN2 exhibits an apical pattern in root epidermal cells. This demonstrates the simultaneous presence of multiple polarity mechanisms in those cells (Wisniewska et al., 2006). Our results demonstrated that *PpPINA* and *PpPINB* endogenously expressed in the protonemata filaments of *P. patens* present different localization patterns (Figure 4 and Supplemental Figure 4). PpPINA exhibits tip-focused PM

localization, whereas PpPINB can be found more spread out on the PM and in the cytoplasm. This polar localization pattern is thought to drive polar auxin transport from the base of the colony toward the tip of filamentous cells and plays an essential role in *P. patens* development (Thelander et al., 2018). Although the functional importance of this difference remains unclear, it demonstrates that parallel polarity/trafficking mechanisms exist in bryophytes in the same cells to which different PINs can be recruited. This is presumably based on specific sequence-based signals.

Notably, the sequences of the HL regions of PpPINA and PpPINB are 90.31% identical, which suggests that these signals are encoded within the divergent 10%. Further analysis of the differences between PpPINA and PpPINB or AtPIN1 and AtPIN2 would help to identify the sequence signals required for PIN polarity regulation. Although the identity of the sequence-based signals remains unclear, our observations show that cellular polarity mechanisms and PIN sequence-based polarity signals, which are crucial for diverse developmental roles in flowering plants, began diversifying in bryophytes.

Context-specific determinants of PIN polarity

It is well-known from *Arabidopsis* that the same PINs show different localization patterns in different contexts (Vieten et al., 2005). For example, AtPIN2 exhibits an apical localization pattern in epidermal cells, while it is localized on the basal side of young cortex cells (Kleine-Vehn et al., 2008a). The observations imply that different cell types possess specific trafficking pathways for the same PIN protein. In line with this, our results show that endogenous PIN proteins are polarized at the tip of apical cells in filamentous cells (e.g. protonemata in *P. patens*, rhizoids in *M. polymorpha*, and root hairs in *Arabidopsis*) (Figure 4A–4C). Notably, MpPINZ-GFP and AtPIN2-GFP signals were diminished when the rhizoids or root hairs elongated. These data collectively demonstrate that PIN polarity is differentially regulated in different developmental contexts.

In complex tissues with multiple cell layers, unlike in filamentous tissues, the PM-localized MpPINZ-GFP did not show polarity in thalli. However, PpPINA-GFP and AtPIN2-GFP presented polar localization at the apical-basal domain of the cells (Figure 4A–4C). These data demonstrate that PIN polarity and trafficking mechanisms have evolved with specific modifications in different tissues and cell types in angiosperms and bryophytes. This likely reflects different requirements for directional auxin transport in different developmental contexts, and it implies co-evolution of PIN sequence-based signals and cell-type-specific polar sorting and trafficking mechanisms.

Diversification of PIN trafficking and polarity mechanisms during land plant evolution

The core mechanisms for auxin biosynthesis, auxin signaling, and PIN-mediated auxin transport are conserved across land plants (Kato et al., 2018; Sauer and Kleine-Vehn, 2019; Blazquez et al., 2020). However, bryophytes and vascular plants diverged around 450 million years ago and developed different tissue and organ types. It is unclear how conserved PIN polarity regulation is under such drastic changes that occurred during land plant evolution. Our cross-species studies revealed that exogenous PINs, such as *Arabidopsis* PINs expressed in bryophytes and bryophytic PINs expressed in *Arabidopsis*, can traffic to the PM (Figure 5). This suggests that all canonical PINs can be recognized by the general protein transport machinery in other species. When the *Arabidopsis* PINs are ectopically expressed in bryophytes, they fail to form any specific polarity, whereas bryophytic PINs remain in apical-basal domains in *Arabidopsis* epidermal cells (Figure 5). These observations hint towards the evolutionary loss of regulatory motifs required for PIN polarization that are present in bryophytic PINs but absent in *Arabidopsis* PINs. In line with this, the coding sequences of bryophytic PINs are longer than *Arabidopsis* PINs. The extra sequences are positioned in their HL regions, which are the main regulatory regions for PIN polarization. Our study demonstrates that PIN polarity mechanisms have not been conserved throughout plant evolution.

This hypothesis was also verified by the differences in cytoskeleton requirements for PIN polarization between bryophytes and angiosperms. The polarity of bryophytic PINs was disrupted when cytoskeletal networks were depolymerized, whereas *Arabidopsis* AtPIN2 was not affected (Figure 6). The most striking result is the hyperpolarization of PpPINA at the tip when actin filaments are disrupted. We hypothesize that focal exocytosis or endocytosis accounting for PpPINA polarity maintenance may rely on actin enrichment at the tip. This finding suggests a gradual shift in the dependence of PIN polarity and trafficking from the cytoskeleton-dependent pathways toward the cytoskeleton-independent pathways during land plant evolution.

Overall, our results demonstrate that different plant species evolved specialized pathways to deliver PINs and maintain their polarity at the PM. This is likely linked to an increasing repertoire of auxin transport developmental roles adopted with increasing morphological complexity during land plant evolution.

METHODS

Plant growth and transformation

Arabidopsis seeds were surface sterilized and grown on 1/2 Murashige-Skoog (MS) plates. After two days of stratification at 4°C, seedlings were grown under long-day conditions (16 h light, 8 h dark) at 22°C with 100-120 μmol photons $m^{-2}s^{-1}$ of white light. For *P. patens*, all transgenic and WT plants were cultured on standard moss BCD medium plates in a growth chamber at 24°C under long-day conditions (16 h light, 8 h dark) with 35 μmol photons $m^{-2}s^{-1}$ of white light. For *M. polymorpha*, WT and all transgenic plants were cultured on 1/2 B5 plates in a growth chamber under long-day conditions (16 h light, 8 h dark) at 22°C with 50-60 μmol photons $m^{-2}s^{-1}$ of white light-emitting diode lighting.

All transgenic plants used in this study are listed in Supplemental Table 1. *P. patens* transgenic plants expressing *pPINA::PINA-GFP* were generated and verified as reported previously (Viaene et al., 2014). The inducible overexpression *XVE::AtPIN1-GFP*, *XVE::AtPIN2-GFP*, and *XVE::Mp-PINZ-GFP* lines were generated as described in previous studies (Yamada et al., 2016; Tang et al., 2020). In brief, Gransden 2004 WT moss plants were freshly propagated and transformed via polyethylene glycol-mediated transformation (Nishiyama et al., 2000). The transformants were selected under hygromycin (20 μg/ml), and two independent transgenic lines were selected and verified for imaging and analysis.

p35S::MpPINZ-, *AtPIN1-*, and *AtPIN2-GFP M. polymorpha* plants were generated via the *Agrobacterium* transformation method described before (Kubota et al., 2013). In brief, the apical meristem region of each two-week-old Takaragaike-1 thallus was removed and cut into four pieces. After culturing on 1/2 B5 with 1% sucrose agar plates for three days, the cut thalli were transferred to 50 ml 0M51C medium with 200 μM acetosyringone (4′-hydroxy-3′,5′-dimethoxyacetophenone) in 200-ml flasks with 130 rpm agitation and cocultured with agrobacteria (optical density 600 = 1) harboring the target construct for another three days. The transformed thalli were washed and plated on 1/2 B5 plates with proper antibiotic selection. Independent T1 lines were isolated, and G1 lines from independent T1 lines were generated by the subcultivation of single gemmalings, which emerged asexually from a single initial cell (Shimamura, 2016). The next generation of G1, called the G2 generation, was used for analyses.

Arabidopsis transgenic lines bearing bryophytic *PIN-GFPs* under the *AtPIN2* promoter control were generated and used as in a previous study (Zhang et al., 2019). For root imaging, seeds were sown on 1/2 MS medium plates, kept at 4°C for two days, and moved to the growth chamber to culture vertically for another four days.

Plasmid construction

Plasmids and primers for construction and genotype confirmation are listed in Supplemental Table 2. For transgenic moss lines with inducible overexpression, the insertion site of the *GFP* gene into the HL is indicated in Figure 3A, and the *PIN-GFP* regions were amplified from previously generated plasmids, which used the genomic DNA for *PpPINA* and *AtPIN1* and the coding sequence for *MpPINZ* and *AtPIN2* (Zhang et al., 2019). *PIN-GFP* was cloned into the Gateway entry plasmid pENTR/D-TOPO as the manufacturer suggested and subcloned into the pPGX8 vector, which contained a p*35S*-driven β-estradiol-inducible XVE cassette (Nakaoka et al., 2012; Kubo et al., 2013; Floriach-Clark et al., 2021) via a Gateway LR reaction (Invitrogen) according to the manufacturer's recommendation.

To generate *p35S::MpPINZ-*, *AtPIN1-*, and *AtPIN2-GFP* constructs, the same *PIN-GFP* fragments as mentioned above were amplified with the primers listed in Supplemental Table 2. The amplified fragments were cloned into the pENTR/D-TOPO vector (Invitrogen) using the protocol supplied by the manufacturer. Plasmids with target genes were subcloned into the pMpGWB102 vector containing a *35S* promoter (Ishizaki et al., 2015) using a Gateway LR reaction (Invitrogen) according to the manufacturer's recommendation.

Microscopy

Moss protonemata were cultured in glass-bottom dishes covered with BCD agar medium for 6-7 days before microscopy. Live-cell imaging was performed using a Leica SP8X-SMD confocal microscope equipped with a hybrid single-molecule detector (HyD) and an ultrashort pulsed white-light laser (50%; 1 ps at 40-MHz frequency). Leica Application Suite X was used for microscope control, and an HC PL APO CS2 40×/1.20 water immersion objective was used for observing the samples. The following imaging settings were used: scan speed of 400 Hz, resolution of 1024 × 1024 pixels, and standard acquisition mode for the hybrid detector. The time-gating system was activated to avoid autofluorescence emitted by chloroplasts. For the filament growth assay, the imaging dish was supplied with an FM4-64 (Invitrogen) solution for 10-30 min, and a 10× objective lens was used.

Marchantia were observed by picking gemmae from a gemma cup and transferring them into a 24-well plate with 500 μL of liquid 1/2 B5 medium. Gemmae were cultured in the growth chamber for 24 before each sample was transferred to a slide and observed under a Leica Stellaris 8 system with HyD detectors and an ultrashort pulsed white-light laser (70%; 1 ps at 40-MHz frequency). Leica Application Suite X was used for microscope control, and an HC PL APO CS2 40×/1.20 water immersion objective was used for observing the samples. The following settings were used: scan speed of 400 Hz and resolution of 1024 × 1024 pixels. GFP fluorescence was detected by exciting samples with a 488-nm white light laser, and setting the detection range between 500 and 525 nm. The tau-gating model was used to avoid autofluorescence emitted by chloroplasts by harvesting photons with a 1.0- to 10.0-ns lifetime for all *Marchantia* imaging. For the surface section (rhizoid precursor cell observation), a 5-μm-thick section was set using the z section method with auto-optimization spacing to capture rhizoid protrusion.

For *Arabidopsis* root imaging, four-day-old seedlings of each indicated genotype were used for fluorescence imaging. After treatment roots with liquid MS medium supplied with the indicated chemicals, seedlings were carefully mounted on a slide with growth medium and placed into a chambered coverslip (Lab-Tek) for imaging. For root hair imaging, a 3-μm Z projection image with 1-μm steps was taken around the medium plane of the root hair. All fluorescence imaging was performed using a laser-scanning confocal microscope (Carl Zeiss LSM800, 20× air lens). Fluorescence from GFP was detected using a 488-nm excitation source and 495-545-nm emission filter.

Image quantification

All images were analyzed using Fiji (ImageJ; https://imagej.net/software/fiji/). For the polarization patterns at the tip of filaments in *P. patens*, a line with 5-pixel thickness was plotted along the PM as depicted in Figures 4 and 5. Representative images for the DMSO control and drug treatments, obtained using the same imaging settings, were used to draw the line. The mean intensity along the line is shown. For the moss phenotype analysis, moss expressing the inducible *PIN-GFP* construct was cultivated in the imaging dish for five days followed by 1 μM β-estradiol induction for another three days. The cell outlines were stained with FM4-64 for 10-30 min. The line drawing function in Fiji was used to measure the length of the subapical cell, and the line is depicted between the middle points of the two cell division planes. The angle measurement function was applied to examine the angle between the first cell division plane and horizontal cell outline for division angle measurements.

Genomic DNA (gDNA) isolation

The gDNA of transformants was isolated by cetyltrimethylammonium bromide gDNA extraction (Schlink and Reski, 2002). In brief, moss tissues were harvested from one full plate and ground in liquid nitrogen. The ground tissues were then mixed and incubated with cetyltrimethylammonium bromide buffer, followed by the addition of chloroform. After centrifugation, the supernatant was collected and precipitated with isopropanol at -20°C for 1 h.

Pharmacological treatments

Ory (Sigma) and LatB (Sigma) treatments were used to depolymerize microtubules and actin filaments, respectively, in plant cells. The concentration and duration of each treatment for each plant species are described in the main text, and the conditions we used have been shown to efficiently depolymerize cytoskeletal networks in multiple species (Baskin et al., 1994; Baluska et al., 2001; Oda et al., 2009; Vidali et al., 2009; Glanc et al., 2019). For *P. patens* and *Arabidopsis*, we used 20 μM LatB or 5 μM Ory for 4 h. The chemicals were diluted in liquid BCD medium and applied to the imaging dishes before imaging.

For *M. polymorpha*, G2 gemmae from transgenic plants were transferred into each well of a 24-well plate containing 500 μL liquid 1/2 B5 medium and cultured in the growth chamber for 16 h. The chemicals were diluted directly into the medium prior to imaging at the indicated time. Based on previous studies, the concentrations of Ory (Era et al., 2013) and LatB (Otani et al., 2018) were selected. The gemmae were treated with 2 μM LatB or 10 μM Ory for 2 h.

For *Arabidopsis*, four-day-old seedlings were submerged in liquid MS medium supplemented with chemical inhibitors and transferred to a separate agar medium for imaging. 0.1% DMSO (Duchefa, 10 mM dimethyl sulfoxide) was used as a control for all treatments.

P. patens auxin export assay

The auxin export assay performed with transgenic moss plants was modified based on the protocol developed for *Arabidopsis* seedlings (Lewis and Muday, 2009). In brief, seven-day-old fresh tissues were transferred to liquid BCDAT growth medium containing 1 μM β-estradiol for four days with gentle shaking. This induction step was followed by treatment with 10 nM ^{3}H-IAA for 24 h. The radioactive tissues were then washed twice with sterile H_2O and cultivated in fresh BCDAT medium for another 24 h. The cultivated medium was then collected and mixed with ScintiVerse BD cocktail (Fisher, SX18-4) at a 1:30 (v:v) ratio. Auxin export was measured using a scintillation counter (Beckman Coulter Genomics, LS6500).

M. polymorpha thallus growth assay

G2 gemmae were transferred onto 1/2 B5 agar plates and grown for 10 days. Gemmae were imaged under a dissection microscope (SZN71, LiWeng, Taiwan) with a charge-coupled device camera (Polychrome M, LiWeng, Taiwan). To measure the vertical growth angle, an agar cube with an individual plant was cut out from the plate and placed in the middle of a slide. The slide was put on the surface of a laminar flow hood at a fixed distance from the edge, and images were taken using an HTC U11 cell phone camera. The growth angle was measured using ImageJ (https://imagej.net/software/fiji/).

Phylogenetic analysis

The phylogenetic analysis for full-length amino acid sequences of all examined PINs was carried out using MEGA X (Kumar et al., 2018), and the results were imported into interactive tree of life (iTOL) (https://itol.embl.de/) for visual analysis. The evolutionary history was inferred by using the maximum likelihood method and Jones-Taylor-Thornton matrix-based model with default settings (Jones et al., 1992). The alignment and identity index were produced using the online CLUSTAL alignment program with default settings.

SUPPLEMENTAL INFORMATION

Supplemental information is available at *Plant Communications Online*.

FUNDING

This work was supported by the European Research Council Advanced Grant (ETAP-742985 to H.T. and J.F.) and by the Ministry of Science and Technology (grant 110-2636-B-005-001 to K.-J.L.).

AUTHOR CONTRIBUTIONS

H.T., K.-J.L., and J.F. initiated and designed the experiments. Y.Z. and Y.-L.C. provided key constructs and materials. H.T. performed and analyzed all *P. patens*- and *Arabidopsis*-related experiments with the help of S.-L.T., and K.-J.L. performed and analyzed all *M. polymorpha*-related experiments. H.T. and K.-J.L. wrote the manuscript with the supervision of J.F.

ACKNOWLEDGMENTS

The authors would like to thank Dr. A. Johnson and Ms. Alexandra Mally for careful proofreading. We also thank Dr. T. Kohchi for sharing the pMpGWB102 vector (Addgene 68556; http://n2t.net/addgene:68556; RRID:Addgene_68556). No conflict of interest is declared.

Received: January 6, 2023
Revised: July 3, 2023
Accepted: July 30, 2023
Published: July 31, 2023

REFERENCES

Abas, L., Benjamins, R., Malenica, N., Paciorek, T., Wiśniewska, J., Moulinier-Anzola, J.C., Sieberer, T., Friml, J., and Luschnig, C. (2006). Intracellular trafficking and proteolysis of the Arabidopsis auxin-efflux facilitator PIN2 are involved in root gravitropism. Nat. Cell Biol. **8**:249–256. https://doi.org/10.1038/ncb1369.

Adamowski, M., and Friml, J. (2015). PIN-dependent auxin transport: action, regulation, and evolution. Plant Cell **27**:20–32. https://doi.org/10.1105/tpc.114.134874.

Baluska, F., Jasik, J., Edelmann, H.G., Salajová, T., and Volkmann, D. (2001). Latrunculin B-induced plant dwarfism: Plant cell elongation is F-actin-dependent. Dev. Biol. **231**:113–124. https://doi.org/10.1006/dbio.2000.0115.

Barbosa, I.C.R., Hammes, U.Z., and Schwechheimer, C. (2018). Activation and Polarity Control of PIN-FORMED Auxin Transporters by Phosphorylation. Trends Plant Sci. **23**:523–538. https://doi.org/10.1016/j.tplants.2018.03.009.

Baskin, T.I., Wilson, J.E., Cork, A., and Williamson, R.E. (1994). Morphology and microtubule organization in Arabidopsis roots exposed to oryzalin or taxol. Plant Cell Physiol. **35**:935–942.

Bennett, T.A., Liu, M.M., Aoyama, T., Bierfreund, N.M., Braun, M., Coudert, Y., Dennis, R.J., O'Connor, D., Wang, X.Y., White, C.D., et al. (2014). Plasma membrane-targeted PIN proteins drive shoot development in a moss. Curr. Biol. **24**:2776–2785. https://doi.org/10.1016/j.cub.2014.09.054.

Blázquez, M.A., Nelson, D.C., and Weijers, D. (2020). Evolution of Plant Hormone Response Pathways. Annu. Rev. Plant Biol. **71**:327–353. https://doi.org/10.1146/annurev-arplant-050718-100309.

Doyle, S.M., Haeger, A., Vain, T., Rigal, A., Viotti, C., Łangowska, M., Ma, Q., Friml, J., Raikhel, N.V., Hicks, G.R., and Robert, S. (2015). An early secretory pathway mediated by GNOM-LIKE 1 and GNOM is essential for basal polarity establishment in Arabidopsis thaliana. Proc. Natl. Acad. Sci. USA **112**:E806–E815. https://doi.org/10.1073/pnas.1424856112.

Era, A., Kutsuna, N., Higaki, T., Hasezawa, S., Nakano, A., and Ueda, T. (2013). Microtubule stability affects the unique motility of F-actin in Marchantia polymorpha. J. Plant Res. **126**:113–119. https://doi.org/10.1007/s10265-012-0496-4.

Floriach-Clark, J., Tang, H., and Willemsen, V. (2021). Mosses: Accessible Systems for Plant Development Studies. In Model Organisms in Plant Genetics, *Ch. 3*, Y.A. Ibrokhim, ed. (IntechOpen: Rijeka). https://doi.org/10.5772/intechopen.100535.

Friml, J. (2021). Fourteen Stations of Auxin. Cold Spring Harbor Perspect. Biol. **14**:a039859. https://doi.org/10.1101/cshperspect.a039859.

Friml, J., Yang, X., Michniewicz, M., Weijers, D., Quint, A., Tietz, O., Benjamins, R., Ouwerkerk, P.B.F., Ljung, K., Sandberg, G., et al. (2004). A PINOID-dependent binary switch in apical-basal PIN polar targeting directs auxin efflux. Science **306**:862–865. https://doi.org/10.1126/science.1100618.

Geldner, N., Friml, J., Stierhof, Y.D., Jürgens, G., and Palme, K. (2001). Auxin transport inhibitors block PIN1 cycling and vesicle trafficking. Nature **413**:425–428. https://doi.org/10.1038/35096571.

Glanc, M., Fendrych, M., and Friml, J. (2018). Mechanistic framework for cell-intrinsic re-establishment of PIN2 polarity after cell division. Nat. Plants **4**:1082–1088. https://doi.org/10.1038/s41477-018-0318-3.

Glanc, M., Fendrych, M., and Friml, J. (2019). PIN2 Polarity Establishment in Arabidopsis in the Absence of an Intact Cytoskeleton. Biomolecules **9**:222. https://doi.org/10.3390/biom9060222.

Ishizaki, K., Nishihama, R., Ueda, M., Inoue, K., Ishida, S., Nishimura, Y., Shikanai, T., and Kohchi, T. (2015). Development of Gateway Binary Vector Series with Four Different Selection Markers for the

Liverwort Marchantia polymorpha. PLoS One **10**, e0138876. https://doi.org/10.1371/journal.pone.0138876.

Jones, D.T., Taylor, W.R., and Thornton, J.M. (1992). The rapid generation of mutation data matrices from protein sequences. Comput. Appl. Biosci. **8**:275–282. https://doi.org/10.1093/bioinformatics/8.3.275.

Jumper, J., Evans, R., Pritzel, A., Green, T., Figurnov, M., Ronneberger, O., Tunyasuvunakool, K., Bates, R., Žídek, A., Potapenko, A., et al. (2021). Highly accurate protein structure prediction with AlphaFold. Nature **596**:583–589. https://doi.org/10.1038/s41586-021-03819-2.

Kato, H., Nishihama, R., Weijers, D., and Kohchi, T. (2018). Evolution of nuclear auxin signaling: lessons from genetic studies with basal land plants. J. Exp. Bot. **69**:291–301. https://doi.org/10.1093/jxb/erx267.

Kato, H., Kouno, M., Takeda, M., Suzuki, H., Ishizaki, K., Nishihama, R., and Kohchi, T. (2017). The Roles of the Sole Activator-Type Auxin Response Factor in Pattern Formation of Marchantia polymorpha. Plant Cell Physiol. **58**:1642–1651. https://doi.org/10.1093/pcp/pcx095.

Kleine-Vehn, J., Langowski, L., Wisniewska, J., Dhonukshe, P., Brewer, P.B., and Friml, J. (2008a). Cellular and molecular requirements for polar PIN targeting and transcytosis in plants. Mol. Plant **1**:1056–1066. https://doi.org/10.1093/mp/ssn062.

Kleine-Vehn, J., Dhonukshe, P., Sauer, M., Brewer, P.B., Wiśniewska, J., Paciorek, T., Benková, E., and Friml, J. (2008b). ARF GEF-dependent transcytosis and polar delivery of PIN auxin carriers in Arabidopsis. Curr. Biol. **18**:526–531. https://doi.org/10.1016/j.cub.2008.03.021.

Kleine-Vehn, J., Wabnik, K., Martinière, A., Łangowski, Ł., Willig, K., Naramoto, S., Leitner, J., Tanaka, H., Jakobs, S., Robert, S., et al. (2011). Recycling, clustering, and endocytosis jointly maintain PIN auxin carrier polarity at the plasma membrane. Mol. Syst. Biol. **7**:540. https://doi.org/10.1038/msb.2011.72.

Krecek, P., Skupa, P., Libus, J., Naramoto, S., Tejos, R., Friml, J., and Zazímalová, E. (2009). The PIN-FORMED (PIN) protein family of auxin transporters. Genome Biol. **10**:249. https://doi.org/10.1186/gb-2009-10-12-249.

Kubo, M., Imai, A., Nishiyama, T., Ishikawa, M., Sato, Y., Kurata, T., Hiwatashi, Y., Reski, R., and Hasebe, M. (2013). System for stable beta-estradiol-inducible gene expression in the moss Physcomitrella patens. PLoS One **8**, e77356. https://doi.org/10.1371/journal.pone.0077356.

Kubota, A., Ishizaki, K., Hosaka, M., and Kohchi, T. (2013). Efficient Agrobacterium-mediated transformation of the liverwort Marchantia polymorpha using regenerating thalli. Biosci. Biotechnol. Biochem. **77**:167–172. https://doi.org/10.1271/bbb.120700.

Kumar, S., Stecher, G., Li, M., Knyaz, C., and Tamura, K. (2018). MEGA X: Molecular Evolutionary Genetics Analysis across Computing Platforms. Mol. Biol. Evol. **35**:1547–1549. https://doi.org/10.1093/molbev/msy096.

Lavy, M., Prigge, M.J., Tao, S., Shain, S., Kuo, A., Kirchsteiger, K., and Estelle, M. (2016). Constitutive auxin response in Physcomitrella reveals complex interactions between Aux/IAA and ARF proteins. Elife **5**, e13325. https://doi.org/10.7554/eLife.13325.

Lewis, D.R., and Muday, G.K. (2009). Measurement of auxin transport in Arabidopsis thaliana. Nat. Protoc. **4**:437–451. https://doi.org/10.1038/nprot.2009.1.

Leyser, O. (2018). Auxin Signaling. Plant Physiol. **176**:465–479. https://doi.org/10.1104/pp.17.00765.

Li, R., and Gundersen, G.G. (2008). Beyond polymer polarity: how the cytoskeleton builds a polarized cell. Nat. Rev. Mol. Cell Biol. **9**:860–873. https://doi.org/10.1038/nrm2522.

Michniewicz, M., Zago, M.K., Abas, L., Weijers, D., Schweighofer, A., Meskiene, I., Heisler, M.G., Ohno, C., Zhang, J., Huang, F., et al. (2007). Antagonistic regulation of PIN phosphorylation by PP2A and PINOID directs auxin flux. Cell **130**:1044–1056. https://doi.org/10.1016/j.cell.2007.07.033.

Mockaitis, K., and Estelle, M. (2008). Auxin receptors and plant development: a new signaling paradigm. Annu. Rev. Cell Dev. Biol. **24**:55–80. https://doi.org/10.1146/annurev.cellbio.23.090506.123214.

Nakaoka, Y., Miki, T., Fujioka, R., Uehara, R., Tomioka, A., Obuse, C., Kubo, M., Hiwatashi, Y., and Goshima, G. (2012). An inducible RNA interference system in Physcomitrella patens reveals a dominant role of augmin in phragmoplast microtubule generation. Plant Cell **24**:1478–1493. https://doi.org/10.1105/tpc.112.098509.

Narasimhan, M., Johnson, A., Prizak, R., Kaufmann, W.A., Tan, S., Casillas-Pérez, B., and Friml, J. (2020). Evolutionarily unique mechanistic framework of clathrin-mediated endocytosis in plants. Elife **9**:e52067. https://doi.org/10.7554/eLife.52067.

Narasimhan, M., Gallei, M., Tan, S., Johnson, A., Verstraeten, I., Li, L., Rodriguez, L., Han, H., Himschoot, E., Wang, R., et al. (2021). Systematic analysis of specific and nonspecific auxin effects on endocytosis and trafficking. Plant Physiol. **186**:1122–1142. https://doi.org/10.1093/plphys/kiab134.

Nishiyama, T., Hiwatashi, Y., Sakakibara, I., Kato, M., and Hasebe, M. (2000). Tagged mutagenesis and gene-trap in the moss, Physcomitrella patens by shuttle mutagenesis. DNA Res. **7**:9–17. https://doi.org/10.1093/dnares/7.1.9.

Oda, Y., Hirata, A., Sano, T., Fujita, T., Hiwatashi, Y., Sato, Y., Kadota, A., Hasebe, M., and Hasezawa, S. (2009). Microtubules regulate dynamic organization of vacuoles in Physcomitrella patens. Plant Cell Physiol. **50**:855–868. https://doi.org/10.1093/pcp/pcp031.

Omelyanchuk, N.A., Kovrizhnykh, V.V., Oshchepkova, E.A., Pasternak, T., Palme, K., and Mironova, V.V. (2016). A detailed expression map of the PIN1 auxin transporter in Arabidopsis thaliana root. BMC Plant Biol. **16** (*Suppl 1*):5. https://doi.org/10.1186/s12870-015-0685-0.

Otani, K., Ishizaki, K., Nishihama, R., Takatani, S., Kohchi, T., Takahashi, T., and Motose, H. (2018). An evolutionarily conserved NIMA-related kinase directs rhizoid tip growth in the basal land plant Marchantia polymorpha. Development **145**:dev154617. https://doi.org/10.1242/dev.154617.

Pettersen, E.F., Goddard, T.D., Huang, C.C., Meng, E.C., Couch, G.S., Croll, T.I., Morris, J.H., and Ferrin, T.E. (2021). UCSF ChimeraX: Structure visualization for researchers, educators, and developers. Protein Sci. **30**:70–82. https://doi.org/10.1002/pro.3943.

Rensing, S.A., Goffinet, B., Meyberg, R., Wu, S.Z., and Bezanilla, M. (2020). The Moss Physcomitrium (Physcomitrella) patens: A Model Organism for Non-Seed Plants. Plant Cell **32**:1361–1376. https://doi.org/10.1105/tpc.19.00828.

Sauer, M., and Kleine-Vehn, J. (2019). PIN-FORMED and PIN-LIKES auxin transport facilitators. Development **146**, dev.168088. https://doi.org/10.1242/dev.168088.

Schlink, K., and Reski, R. (2002). Preparing high-quality DNA from moss (Physcomitrella patens). Plant Mol. Biol. Rep. **20**:423.

Shimamura, M. (2016). Marchantia polymorpha: Taxonomy, Phylogeny and Morphology of a Model System. Plant Cell Physiol. **57**:230–256. https://doi.org/10.1093/pcp/pcv192.

Skokan, R., Medvecká, E., Viaene, T., Vosolsobě, S., Zwiewka, M., Müller, K., Skůpa, P., Karady, M., Zhang, Y., Janacek, D.P., et al. (2019). PIN-driven auxin transport emerged early in streptophyte evolution. Nat. Plants **5**:1114–1119. https://doi.org/10.1038/s41477-019-0542-5.

Smit, M.E., and Weijers, D. (2015). The role of auxin signaling in early embryo pattern formation. Curr. Opin. Plant Biol. **28**:99–105. https://doi.org/10.1016/j.pbi.2015.10.001.

Sukumar, P., Edwards, K.S., Rahman, A., Delong, A., and Muday, G.K. (2009). PINOID kinase regulates root gravitropism through modulation of PIN2-dependent basipetal auxin transport in Arabidopsis. Plant Physiol. **150**:722–735. https://doi.org/10.1104/pp.108.131607.

Tang, H., Duijts, K., Bezanilla, M., Scheres, B., Vermeer, J.E.M., and Willemsen, V. (2020). Geometric cues forecast the switch from two- to three-dimensional growth in Physcomitrella patens. New Phytol. **225**:1945–1955. https://doi.org/10.1111/nph.16276.

Tao, S., and Estelle, M. (2018). Mutational studies of the Aux/IAA proteins in Physcomitrella reveal novel insights into their function. New Phytol. **218**:1534–1542. https://doi.org/10.1111/nph.15039.

Thelander, M., Landberg, K., and Sundberg, E. (2018). Auxin-mediated developmental control in the moss Physcomitrella patens. J. Exp. Bot. **69**:277–290. https://doi.org/10.1093/jxb/erx255.

Ung, K.L., Winkler, M., Schulz, L., Kolb, M., Janacek, D.P., Dedic, E., Stokes, D.L., Hammes, U.Z., and Pedersen, B.P. (2022). Structures and mechanism of the plant PIN-FORMED auxin transporter. Nature **609**:605–610. https://doi.org/10.1038/s41586-022-04883-y.

Vanneste, S., and Friml, J. (2009). Auxin: a trigger for change in plant development. Cell **136**:1005–1016. https://doi.org/10.1016/j.cell.2009.03.001.

Viaene, T., Delwiche, C.F., Rensing, S.A., and Friml, J. (2013). Origin and evolution of PIN auxin transporters in the green lineage. Trends Plant Sci. **18**:5–10. https://doi.org/10.1016/j.tplants.2012.08.009.

Viaene, T., Landberg, K., Thelander, M., Medvecka, E., Pederson, E., Feraru, E., Cooper, E.D., Karimi, M., Delwiche, C.F., Ljung, K., et al. (2014). Directional auxin transport mechanisms in early diverging land plants. Curr. Biol. **24**:2786–2791. https://doi.org/10.1016/j.cub.2014.09.056.

Vidali, L., Rounds, C.M., Hepler, P.K., and Bezanilla, M. (2009). Lifeact-mEGFP reveals a dynamic apical F-actin network in tip growing plant cells. PLoS One **4**, e5744. https://doi.org/10.1371/journal.pone.0005744.

Vieten, A., Vanneste, S., Wisniewska, J., Benková, E., Benjamins, R., Beeckman, T., Luschnig, C., and Friml, J. (2005). Functional redundancy of PIN proteins is accompanied by auxin-dependent cross-regulation of PIN expression. Development **132**:4521–4531. https://doi.org/10.1242/dev.02027.

Wisniewska, J., Xu, J., Seifertová, D., Brewer, P.B., Ruzicka, K., Blilou, I., Rouquié, D., Benková, E., Scheres, B., and Friml, J. (2006). Polar PIN localization directs auxin flow in plants. Science **312**:883. https://doi.org/10.1126/science.1121356.

Yamada, M., Miki, T., and Goshima, G. (2016). Imaging Mitosis in the Moss Physcomitrella patens. Methods Mol. Biol. **1413**:263–282. https://doi.org/10.1007/978-1-4939-3542-0_17.

Yang, Z., Xia, J., Hong, J., Zhang, C., Wei, H., Ying, W., Sun, C., Sun, L., Mao, Y., Gao, Y., et al. (2022). Structural insights into auxin recognition and efflux by Arabidopsis PIN1. Nature **609**:611–615. https://doi.org/10.1038/s41586-022-05143-9.

Yu, Z., Zhang, F., Friml, J., and Ding, Z. (2022). Auxin signaling: Research advances over the past 30 years. J. Integr. Plant Biol. **64**:371–392. https://doi.org/10.1111/jipb.13225.

Zhang, J., Nodzynski, T., Pencík, A., Rolcík, J., and Friml, J. (2010). PIN phosphorylation is sufficient to mediate PIN polarity and direct auxin transport. Proc. Natl. Acad. Sci. USA **107**:918–922. https://doi.org/10.1073/pnas.0909460107.

Zhang, Y., Xiao, G., Wang, X., Zhang, X., and Friml, J. (2019). Evolution of fast root gravitropism in seed plants. Nat. Commun. **10**:3480. https://doi.org/10.1038/s41467-019-11471-8.

Zourelidou, M., Absmanner, B., Weller, B., Barbosa, I.C.R., Willige, B.C., Fastner, A., Streit, V., Port, S.A., Colcombet, J., de la Fuente van Bentem, S., et al. (2014). Auxin efflux by PIN-FORMED proteins is activated by two different protein kinases, D6 PROTEIN KINASE and PINOID. Elife **3**, e02860. https://doi.org/10.7554/eLife.02860.

Zwiewka, M., Bilanovičová, V., Seifu, Y.W., and Nodzyński, T. (2019). The Nuts and Bolts of PIN Auxin Efflux Carriers. Front. Plant Sci. **10**:985. https://doi.org/10.3389/fpls.2019.00985.

Plant Communications, Volume *5*

Supplemental information

Divergence of trafficking and polarization mechanisms for PIN auxin transporters during land plant evolution

Han Tang, Kuan-Ju Lu, YuZhou Zhang, You-Liang Cheng, Shih-Long Tu, and Jiří Friml

Supplemental Information

Divergence of trafficking and polarization mechanisms for PIN auxin transporters during land plant evolution

Han Tang[1,5,6], Kuan-Ju Lu[2,6], YuZhou Zhang[3], You-Liang Cheng[4], Shih-Long Tu[4], and Jiří Friml[1,*]

1. Institute of Science and Technology Austria (ISTA), Am Campus 1, 3400 Klosterneuburg, Austria
2. Graduate Institute of Biotechnology, National Chung Hsing University, No. 145, Xingda Rd., South Dist., Taichung 40227, Taiwan, R.O.C.
3. College of Life Sciences, Northwest A&F University, Shaanxi, Yangling, China.
4. Institute of Plant and Microbial Biology, Academia Sinica, 128 Sec. 2, Academia Rd, Nankang, Taipei 11529, Taiwan, R.O.C.
5. Current address: Graduate Institute of Biochemistry, National Chung Hsing University, No. 145, Xingda Rd., South Dist., Taichung 40227, Taiwan, R.O.C.

6. These authors contribute equally to this work.

* Correspondence: jiri.friml@ist.ac.at, +43 2243 9000 5401

The supplemental file includes 9 supplemental figures and 2 supplemental tables

Supplemental figure 1. Identity index of all selected PINs compared to AtPIN1.

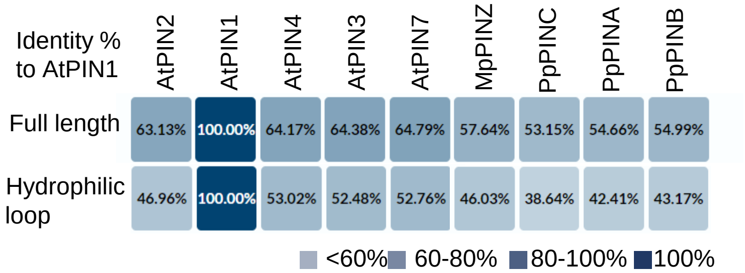

Supplemental figure 1. Identity index of all selected PINs compared to AtPIN1.

Alignment of full length sequence and hydrophilic loop region for canonical PINs from *Physcomitrium patens* (Pp)*, Marchantia polymorpha* (Mp), and *Arabidopsis thaliana* (At).

Supplemental figure 2. PIN structures predicted by Alphafold2 algorism.

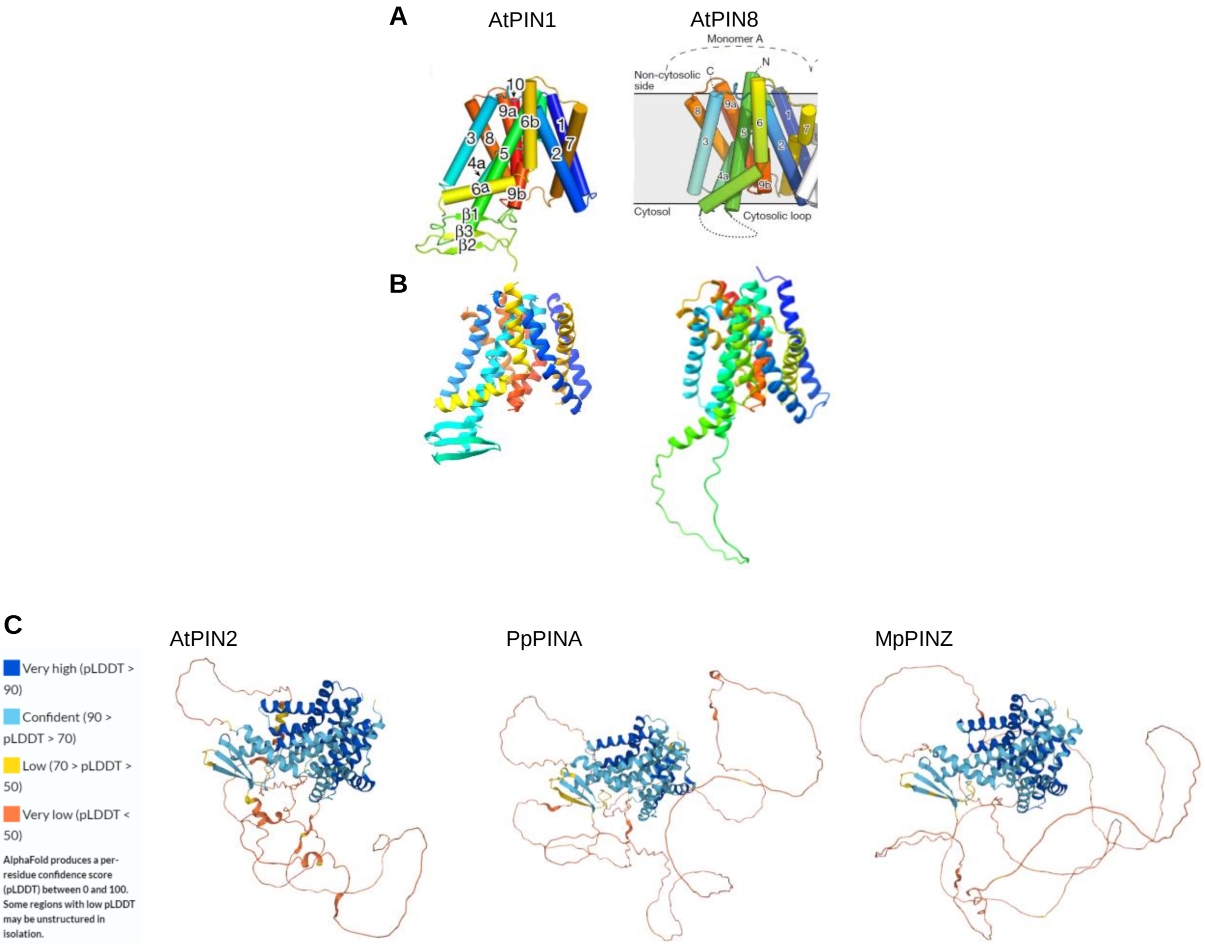

Supplemental figure 2. PIN structures predicted by Alphafold2 algorism.

(A) Overall structures of AtPIN1 and AtPIN8 monomer modified from Ung *et al*. and Yang *et al*. (Ung *et al.,* 2022; Yang *et al.,* 2022)

(B) Predicted structures of AtPIN1 and AtPIN8 by AlphaFold2.

(C) Predicted structures of AtPIN2, PpPINA, and MpPINZ with the confidence score provided by AlphaFold2.

Supplemental figure 3. Genotype confirmation of transgenic lines.

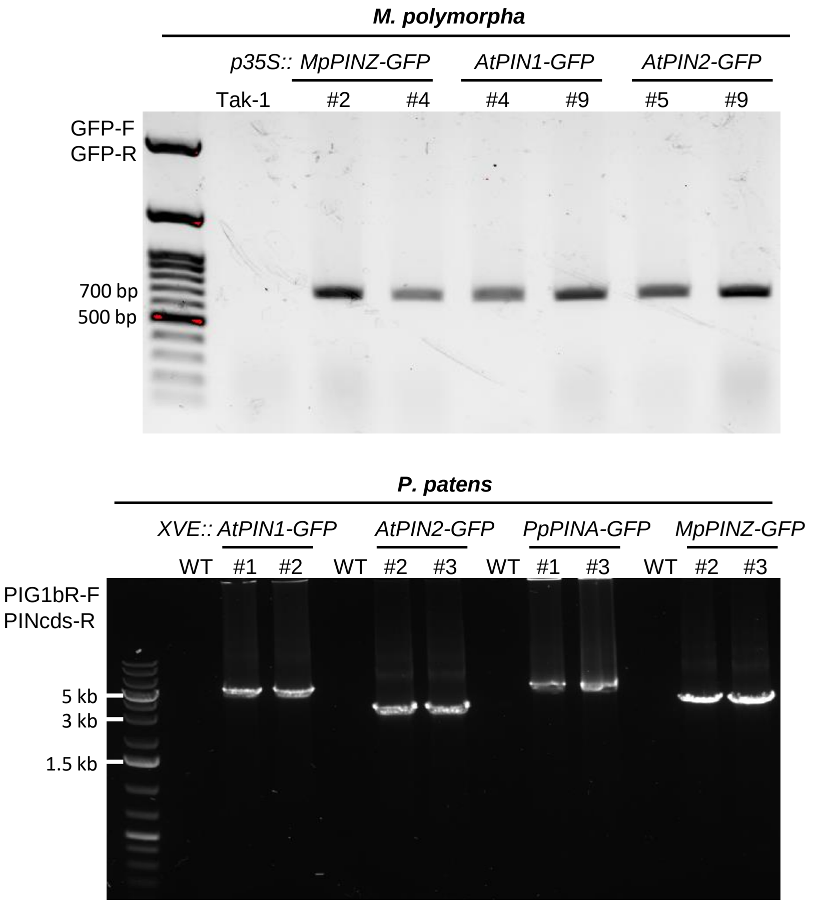

Supplemental figure 3. Confirmation of transgenic lines.

Genomic DNAs extracted from indicated transgenic lines were used as templates for PCR reactions. Primer sets used in the PCR were indicated in the left, and the primer sequences were shown in supplemental table. Two independent lines for each construct were confirmed.

Supplemental figure 4. PpPINA is evenly distributed on the plasma membrane in moss leaves near the base.

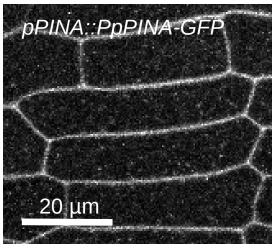

Supplemental figure 4. PpPINA is evenly distributed on the plasma membrane in moss leaves near the base.

As previously reported, PpPINA-GFP showed symmetric distribution in gametophytic leaves. (Viaene *et al.,* 2014).

Supplemental figure 5. PpPINB is evenly distributed on the plasma membrane with a high cytosolic signal in moss protonema cells.

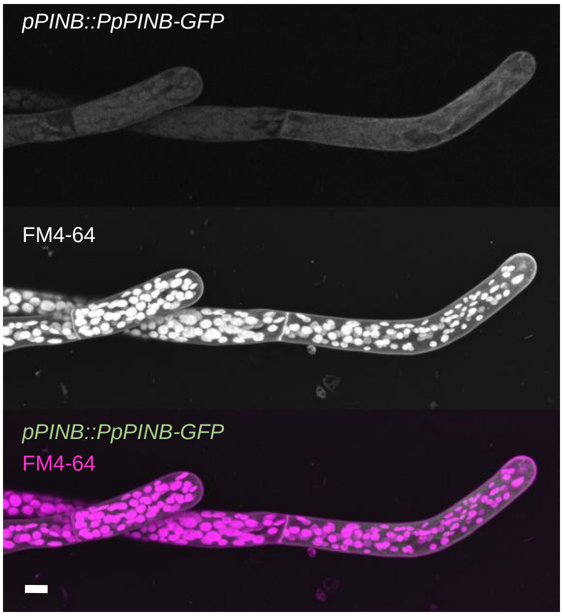

Supplemental figure 5. PpPINB is evenly distributed on the plasma membrane with a high cytosolic signal in moss protonema cells.

PpPINB-GFP is driven by its endogenous promoter and stained with FM4-64 for cell outline imaging. Scale bar = 10 µm.

Supplemental figure 6. MpPINZ-GFP signal is diminished in most elongated rhizoids.

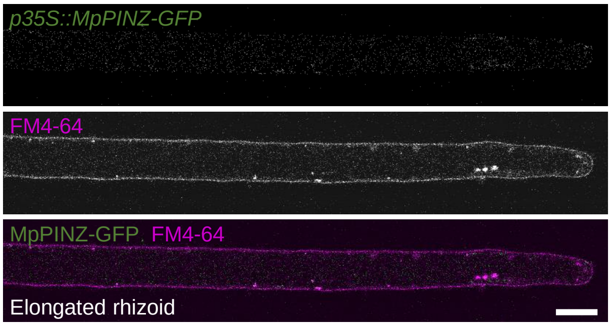

Supplemental figure 6. MpPINZ-GFP signal is diminished in most elongated rhizoids.

Elongated rhizoids were observed under the same imaging conditions as used in Figure 5B. The tissues were stained with FM4-64 for 10-30 minutes prior to imaging. Scale bar = 10 µm.

Supplemental figure 7. AtPIN1-GFP and AtPIN2-GFP show similar localization patterns under shorter induction time in moss protonema cells.

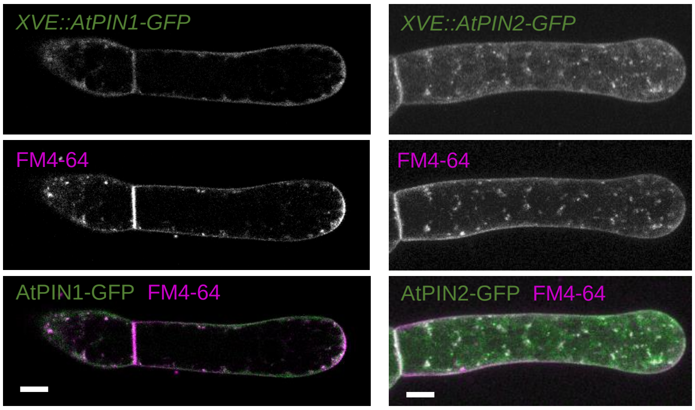

Supplemental figure 7. AtPIN1-GFP and AtPIN2-GFP show similar localization patterns under shorter induction time in moss protonema cells.

Moss tissues were cultivated in the imaging dish for 6 days and the expression of *AtPIN1-GFP* and *AtPIN2-GFP* were induced for additional 24 or 48 hours with 1 µM β-extradiol, respectively. The tissues were stained with FM4-64 for 10-30 minutes prior to imaging. Scale bar = 10 µm.

Supplemental figure 8. Arabidopsis AtPIN1 is not polarized in Marchantia.

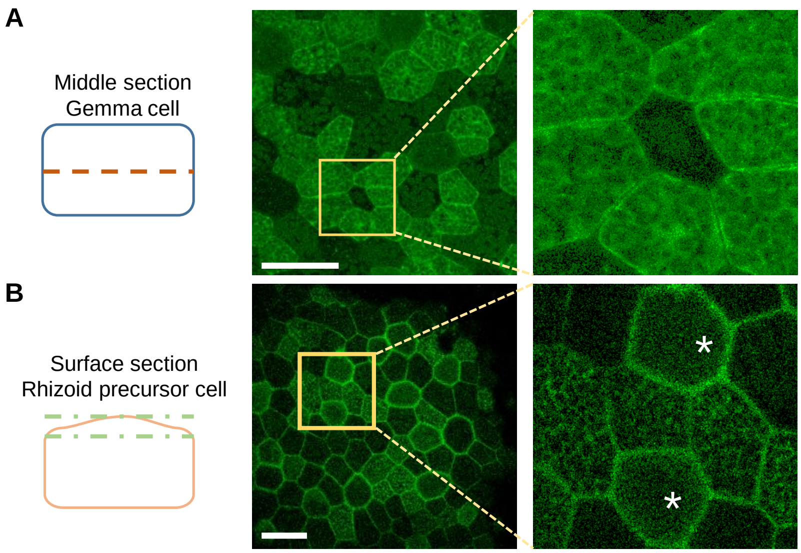

Supplemental figure 8. Arabidopsis AtPIN1 is not polarized in Marchantia.

The localization of AtPIN1-GFP in *M. polymerpha* gemma epidermal cells and protrusion site of initial rhizoids (asterisks). Imaging sections were obtained as illustrated. The AtPIN1-GFP is localized at the plasma membrane and in the cytoplasm of all examined tissues with no apparent puncta signal. Scale bar = 50 mm.

Supplemental figure 9. *MpPINZ-GFP* and *AtPIN1-GFP* driven by MpPINZ endogenous promoter show similar localization patterns in Marchantia.

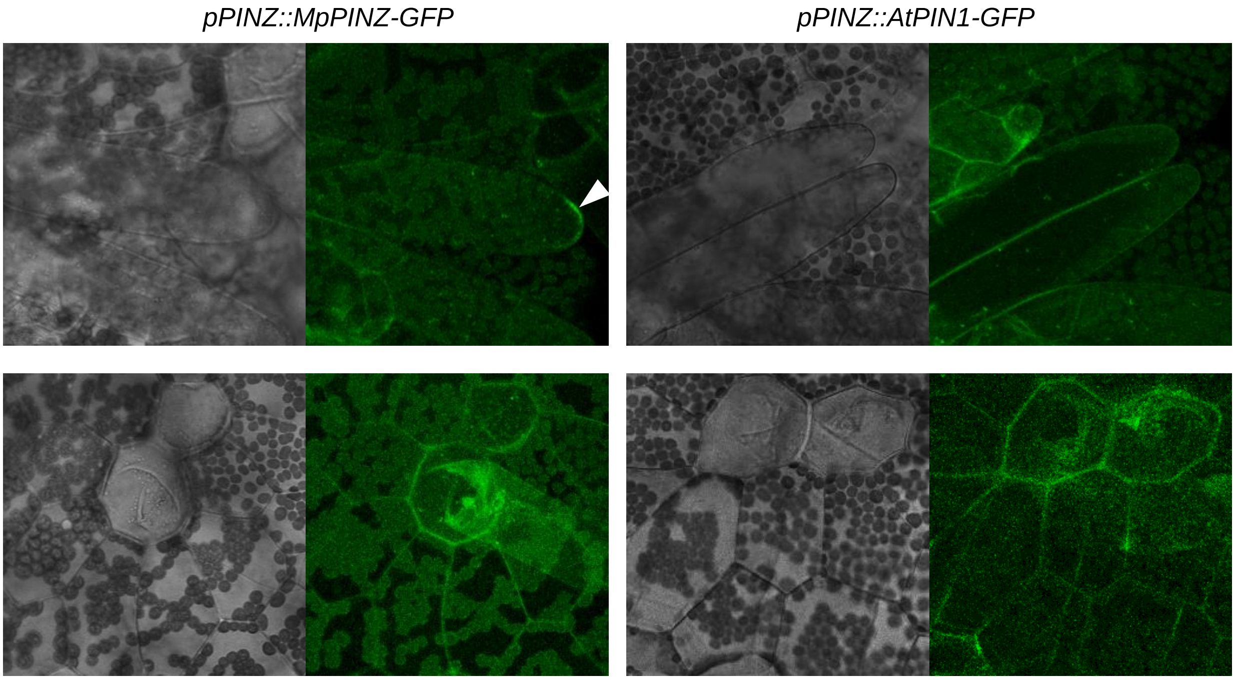

Supplemental figure 9. *MpPINZ-GFP* and *AtPIN1-GFP* driven by *MpPINZ* endogenous promoter show similar localization patterns in Marchantia.

The localization of MpPINZ-GFP and AtPIN1-GFP driven by MpPINZ endogenous promoter. MpPINZ-GFP is polarized at the tip of young rhizoids (white arrowhead), while AtPIN1-GFP is evenly distributed on the PM. Both MpPINZ-GFP and AtPIN1-GFP show PM localization with no polarity in gemma epidermal cells.

References:

1. **Ung KL, Winkler M, Schulz L, Kolb M, Janacek DP, Dedic E, Stokes DL, Hammes UZ, Pedersen BP. 2022.** Structures and mechanism of the plant PIN-FORMED auxin transporter. Nature 609(7927): 605-610.
2. **Viaene T, Landberg K, Thelander M, Medvecka E, Pederson E, Feraru E, Cooper ED, Karimi M, Delwiche CF, Ljung K, et al. 2014.** Directional auxin transport mechanisms in early diverging land plants. Curr Biol 24(23): 2786-2791.
3. **Yang Z, Xia J, Hong J, Zhang C, Wei H, Ying W, Sun C, Sun L, Mao Y, Gao Y, et al. 2022.** Structural insights into auxin recognition and efflux by Arabidopsis PIN1. Nature 609(7927): 611-615.

Supplemental Table 1. Materials used and generated in this study.

Organism	Strain	In use	Source
Physcomitrelium patens	pPINA::PpPINA-GFP	Figure 4, 6, Supplemental Figure 4	Previous study (Viaene et al., 2014)
	pPINB::PpPINB-GFP	Supplemental Figure 5	Previous study (Viaene et al., 2014)
	XVE::PpPINA-GFP	Figure 3, 5	This study
	XVE::AtPIN1-GFP	Figure 3, 5	This study
	XVE::AtPIN2-GFP	Figure 3, Supplemental Figure 7	This study
	XVE::MpPINZ-GFP	Figure 3	This study
Marchantia polymorpha	p35S::MpPINZ-GFP	Figure 3, 4, 5, 6	This study
	p35S::AtPIN1-GFP	Figure 3, 5, Supplemental Figure 8	This study
	p35S::AtPIN2-GFP	Figure 3	This study
	pPINZ::MpPINZ-GFP	Supplemental Figure 9	This study
	pPINZ::AtPIN1-GFP	Supplemental Figure 9	This study
Arabidopsis thaliana	pPIN2::AtPIN2-GFP	Figure 4, 5, 6	Previous study (Zhang et al., 2019)
	pPIN2::PpPINA-GFP	Figure 5	Previous study (Zhang et al., 2019)
	pPIN2::MpPINZ-GFP	Figure 5	Previous study (Zhang et al., 2019)

Supplemental Table 2. Plasmid and primer list used in this study.

**Plasmids**	**Name**	**Description**	**Purpose**
1	pPGX8	inducible XVE promoter, PIG locus	To generate inducible overexpression line in moss
2	pMpGWB102	Binary vector with virus 35S promoter	To generate overexpression line in Marchantia
**Primers**	**Name**	**Sequence**	**Purpose**
1	AtPIN1_TOPO_F	CACC ATGATTACGGCGGCGGACTTC	TOPO cloning
2	AtPIN1cds_R	TCATAGACCCAAGAGAATGTAGTAGAG	TOPO cloning, genotyping PCR
3	AtPIN2_TOPO_F	CACC ATGATCACCGGCAAAGACATGTAC	TOPO cloning
4	AtPIN2cds_R	TTAAAGCCCCAAAAGAACGTAG	TOPO cloning, genotyping PCR
5	PINAcds_R	TCACAGACCAAGTAATATGTAG	genotyping PCR
6	PINZcds_R	TTACAGGCCCAAAAGTACGTAG	genotyping PCR
7	PIGbR_F	GAGTCATCAAATTGAAGTACAAGTAGG	genotyping PCR
8	GFP_F	GCAAGGGCGAGGAGCTGTTC	genotyping PCR
9	GFP_R	GTACAGCTCGTCCATGCCGAG	genotyping PCR
